# Emerging Immune Cell Biomarkers in Primary Membranous Nephropathy: Immune Cell Profiling During Anti-CD20 B Cell-Targeted Therapy

**DOI:** 10.3390/ijms27167233

**Published:** 2026-08-13

**Authors:** Christos Georgopoulos, Eleni Stamellou, Anila Duni, Lefkothea Dova, Georgios Vartholomatos, Ekaterini Siomou, Haralampos Milionis, Evangelia Dounousi

**Affiliations:** 1 Department of Nephrology, University Hospital of Ioannina, University of Ioannina, 45500 Ioannina, Greece; georgopoulosch@gmail.com (C.G.); anikristduni@yahoo.com (A.D.); evangeldou@gmail.com (E.D.); 2 Division of Nephrology and Rheumatology, RWTH Aachen University Hospital, 52074 Aachen, Germany; 3 Laboratory of Hematology, Unit of Molecular Biology, University Hospital of Ioannina, 45500 Ioannina, Greece; ldova@uoi.gr (L.D.); gvarthol@gmail.com (G.V.); 4 Department of Pediatrics, University Hospital of Ioannina, 45500 Ioannina, Greece; eksiomou@yahoo.gr; 5 1st Department of Internal Medicine, University Hospital of Ioannina, 45500 Ioannina, Greece; hmilioni@uoi.gr

**Keywords:** anti-PLA2R1 antibodies, B cells, biomarkers, B cell depletion, membranous nephropathy, rituximab, T regulatory cells, immunological remission, immune monitoring, immunophenotypic profiling

## Abstract

Primary membranous nephropathy (pMN) is an antibody-mediated podocytopathy, most commonly caused by autoantibodies against the M-type phospholipase A2 receptor (PLA2R1). Rituximab (RTX), an anti-CD20 monoclonal antibody, is a first-line treatment for moderate-to-high-risk pMN, inducing partial or complete remission in about 60% of patients within 24 months. However, treatment response varies considerably, and current biomarkers, including anti-PLA2R1 titers and peripheral B cell counts, have limited predictive value for non-response or relapse. Beyond B cell depletion, RTX exerts broader immunomodulatory effects by influencing T cell subsets, monocytes, and natural killer (NK) cells involved in antibody-dependent cellular cytotoxicity. This review examines the peripheral immune cell changes that accompany anti-CD20 therapy and their value as candidate biomarkers. Total CD19+ B cell depletion is the standard pharmacodynamic measure of drug effect but correlates only loosely with clinical outcome. A specific B cell reconstitution profile was associated with pending relapse. Class-switched memory B cells remain depleted during sustained remission, and their premature re-expansion has been associated with subsequent relapse. Regulatory T cells are reduced in active disease and rise within days of infusion in patients who later respond. The systemic inflammation response index, derived from the routine differential count, has been associated with both 6- and 12-month remission. These observations derive from small, mostly single-center cohorts using heterogeneous panels and different RTX regimens. On the available evidence, immune cell profiling cannot yet be recommended for routine disease monitoring, and larger prospective studies with standardized panels are required.

## 1. Introduction

Primary membranous nephropathy (pMN) is an organ-specific autoimmune disorder and remains the most common cause of idiopathic nephrotic syndrome in non-diabetic adults worldwide [1]. The pathophysiological hallmark of the disease is the ongoing formation and deposition of subepithelial immune complexes along the outer side of the glomerular basement membrane (GBM) [1]. The molecular understanding of pMN changed substantially with the identification of specific podocyte target antigens. The discovery that most pMN cases are driven by autoantibodies against M-type phospholipase A2 receptor (PLA2R1) and, less commonly, against THSD7A and NELL-1, established pMN unambiguously as an antibody-mediated autoimmune disease [2,3]. Anti-PLA2R1 and anti-THSD7A antibodies are predominantly of the IgG4 subclass [2,3], and anti-PLA2R1 titers closely correlate with disease activity, clinical severity and long-term renal prognosis [4].

The discovery of PLA2R1 transformed the diagnostic and therapeutic approach to this disease. The principle of a circulating autoantibody serving as a non-invasive diagnostic and activity marker in glomerular disease was established earlier by antineutrophil cytoplasmic antibodies, which were first described in 1985 as both a diagnostic tool and a marker of disease activity in granulomatosis with polyangiitis [5]. What was distinctive about anti-PLA2R1 was that the autoantibody was directed against a defined podocyte antigen, so that the target antigen, the pathogenic autoantibody and a quantitative activity marker became the same molecular entity, permitting non-invasive diagnosis in the appropriate clinical context, longitudinal monitoring of disease activity, and treatment decisions approaching precision medicine [3]. This paradigm is now formalized in the KDIGO 2021 guidelines, which structure the management of pMN around a risk-stratification model that integrates proteinuria, anti-PLA2R1 titer dynamics and renal function [6]. Anti-PLA2R1 titer trajectory under therapy is now considered an immunological surrogate of treatment response that often precedes proteinuria reduction by several months [7].

Despite the strength of this serology-anchored framework, important clinical gaps remain. Proteinuria remains the central outcome measure in pMN and the endpoint on which every randomized trial in the disease has been built. No alternative has displaced it. Its limitation is one of timing rather than of validity: proteinuria reflects the structural consequence of immune injury rather than the immune process itself, so it falls slowly once the immunological process has been controlled, and in longstanding disease with secondary focal segmental glomerulosclerosis it may persist after immunological remission has been achieved [7]. Anti-PLA2R1 testing complements it for precisely this reason, but is informative in 70–80% of pMN cases, while PLA2R1-negative patients require alternative monitoring strategies [6].

Treatment has steadily shifted away from generalized immunosuppression (cyclical alkylating agents combined with high-dose corticosteroids, or calcineurin inhibitors) toward targeted biological therapy. Rituximab (RTX), a chimeric type I anti-CD20 monoclonal antibody, achieves complete or partial remission in approximately 60% of patients at 24 months in randomized trials, with higher rates of up to 70–80% reported in observational cohorts using repeated or extended dosing, and carries a more favorable safety profile than earlier regimens [8,9]. However, approximately 20–40% of patients fail to respond to RTX, and 5–28% of those who achieve remission will subsequently relapse and require retreatment [8,9]. Newer anti-CD20 agents are broadening this approach; in the recently reported phase 3 MAJESTY trial, the glycoengineered, fully humanized anti-CD20 antibody obinutuzumab produced markedly higher complete remission rates than tacrolimus in pMN [10]. Identifying non-responders and future relapsers earlier, ideally before clinical or serological deterioration, would refine the KDIGO risk model and individualize treatment intensity.

Multiparameter flow cytometry offers a way to observe this process at the cellular level, and has been applied in pMN in a small number of cohorts. It resolves B cell, T cell, monocyte and NK-cell subsets that routine clinical tests do not distinguish, and in the setting of anti-CD20 therapy it measures the drug’s target directly. To date, however, this resolution has yielded mechanistic insight rather than clinical utility. No cellular parameter has been shown to improve on proteinuria and anti-PLA2R1 titer in guiding management [6,11]. This review serves a dual purpose: To summarize, subset by subset, what peripheral immune cell profiling has actually demonstrated in pMN, stating for each observation whether it is potentially informative, mechanistically important but clinically unproven, or purely descriptive, and to explain how anti-CD20 therapy remodels each compartment and why depletion does not always produce immunological remission. The scope is deliberately confined to anti-CD20 therapy, the only regimen used in pMN in which the measured cell and the drug target coincide.

### 1.1. Evidence Base and Search Strategy

This is a narrative review. PubMed/MEDLINE, Scopus and Cochrane Central Register of Clinical Trials (CENTRAL) were searched from inception to March 2026 without language restriction, combining the terms “membranous nephropathy” or “primary membranous nephropathy”, “PLA2R” or “anti-PLA2R”, “rituximab”, “obinutuzumab” and “anti-CD20” with “flow cytometry”, “immunophenotyping”, “B-cell subsets”, “memory B cells”, “plasmablasts”, “regulatory T cells”, “regulatory B cells”, “monocyte subsets”, “natural killer cells”, “neutrophil-to-lymphocyte ratio” and “systemic inflammation response index” or “SIRI”. The reference lists of retrieved articles and the KDIGO 2021 glomerular diseases guideline [6] were hand searched. Studies were eligible if they reported peripheral immune cell subsets, or inflammatory indices derived from the routine differential count, in adults with biopsy-proven pMN. Case reports were retained only where they contributed longitudinal kinetic data. Where no evidence in pMN existed, we drew on cohorts in idiopathic nephrotic syndrome, ANCA-associated vasculitis, lupus nephritis, and rheumatoid arthritis, and such statements are identified as extrapolated in the text. Table 1 is restricted to studies performed in pMN and states the treatment regimen of each cohort. The “evidence source” column of Table 2 states, for every parameter, whether the supporting data are direct (RTX-treated pMN), indirect (pMN but non-RTX-treated or cross-sectional) or extrapolated (other disease settings).

### 1.2. Terminology: Marker Categories and Evidence Tiers

Biomarkers are classified here by the clinical question they are used to answer rather than by the cell population measured, because a marker validated for one purpose is not thereby validated for another. Pharmacodynamic markers report drug-target engagement rather than disease biology. Disease-activity markers covary with the intensity of the ongoing autoimmune process. Predictive markers, measured before or early during treatment, identify patients likely to respond to a specific therapy. Prognostic markers relate to outcome irrespective of the treatment given. Relapse markers signal re-emergence of disease after remission has been achieved. A single parameter may fall into more than one category at different time points. Composite indices derived from the routine differential count, such as the systemic inflammation response index, are inflammatory prognostic indices and are distinguished from immunophenotyping by multiparameter flow cytometry. Each parameter in Table 2 is assigned a marker category, an evidence source and a tier: tier A denotes a defined and actionable interpretation in RTX-treated pMN, tier B a plausible role supported by single cohorts or by extrapolation from related diseases, and tier C a parameter that is at present descriptive only.

## 2. The B Lymphocyte Compartment: Pathogenic Drivers and Regulators of Autoimmunity

In pMN, B lymphocytes contribute to disease pathogenesis well beyond their eventual differentiation into autoantibody-secreting plasma cells. B cells are highly effective antigen-presenting cells (APCs) that can internalize and process podocyte antigens (PLA2R1, THSD7A) and present them to specific T cells, thereby promoting the activation and expansion of the helper T cell compartment [24]. They also secrete pro-inflammatory and immunomodulatory cytokines, widening the inflammatory response beyond antibody-mediated injury. A detailed analysis of the B cell compartment requires dividing the total CD19+ population into distinct maturation and functional subsets based on the surface expression of CD27 and IgD [25].

### 2.1. Total B Cells (CD3-CD19+)

The total B cell population is typically identified by the expression of the CD19 lineage marker, along with the absence of the T cell marker CD3 (CD3-CD19+) [24]. This population includes the entire developmental range of peripheral B lymphocytes, from transitional bone marrow emigrants to mature memory cells [25]. In untreated active pMN, the total CD3-CD19+ B cell count is consistently increased relative to healthy controls, although it does not correlate with proteinuria, serum albumin, or eGFR, and is therefore not a reliable measure of disease activity in itself [14].

In the context of RTX treatment, the total CD19+ B cell count is the primary measure of the drug’s immediate pharmacodynamic effect [14]. RTX causes B cell depletion through various mechanisms, including complement-dependent cytotoxicity (CDC), antibody-dependent cellular phagocytosis (ADCP), and antibody-dependent cellular cytotoxicity (ADCC) [26]. RTX can also induce limited direct cell death via CD20 cross-linking, although this is considered a minor contributor for Type I anti-CD20 antibodies compared with the non-apoptotic direct killing characteristic of Type II antibodies such as obinutuzumab [26]. The clinical goal is to achieve complete peripheral B cell depletion, defined as reducing circulating CD19+ cells to less than 1% of all lymphocytes or an absolute count of fewer than 5 cells/µL, usually assessed within the first month after infusion [14]. This threshold is commonly applied in rheumatologic and nephrotic-syndrome studies, although MN-specific thresholds have not been formally validated. These two criteria are, moreover, not interchangeable, as 1% of lymphocytes corresponds to 5 cells/µL only at a total lymphocyte count of 500/µL, and at a normal count of 1500/µL the proportional criterion permits three times as many residual B cells as the absolute one. Because lymphopenia and hemodilution are common in nephrotic patients, percentages and absolute counts may diverge. CD19 rather than CD20 is used for pharmacodynamic monitoring because RTX binding induces epitope masking and CD20 trogocytosis/shaving by phagocytes, rendering standard CD20 staining unreliable post-infusion [8].

Failure to achieve profound depletion or very early B cell repopulation may indicate primary immunologic resistance or inadequate RTX exposure. In severe nephrotic-range proteinuria, urinary loss of intact RTX reduces systemic bioavailability, resulting in sub-therapeutic drug levels and incomplete B cell depletion [27]. Incomplete depletion or rapid early repopulation may also reflect anti-RTX antibodies (ARAs, previously known as HACAs, human anti-chimeric antibodies), which neutralize RTX either by blocking CD20 binding or by accelerating drug clearance [28]. Monitoring CD3-CD19+ kinetics is therefore informative, since it confirms adequate pharmacodynamic response and may also reveal early repopulation patterns that suggest anti-drug antibody development.

### 2.2. Naïve B Cells (CD19+CD27-IgM+IgD+)

Naïve B cells, characterized immunophenotypically as CD19+CD27-IgM+IgD+, are the mature, immunocompetent, yet antigen-inexperienced pool of lymphocytes that originates from the bone marrow to replenish the peripheral immune system [29]. Baseline immunophenotyping in severe pMN has shown a relative expansion of naïve B cells accompanied by a reciprocal reduction in switched and unswitched-memory B cells, consistent with a disturbance of peripheral B cell homeostasis and tolerance checkpoints [11,12]. This likely reflects ongoing recruitment of newly generated naïve cells into the autoimmune response. They encounter podocyte antigens, undergo antigen-driven activation, and leave the naïve compartment to differentiate into memory B cells and plasmablasts in secondary lymphoid tissues [30].

The prognostic importance of the naïve B cell compartment becomes clear during the repopulation phase after successful RTX-induced depletion. As RTX effects wane, typically between 6 and 12 months post-infusion, the B cell compartment begins to reconstitute. In patients who achieve and maintain long-term clinical remission, this reconstitution is largely characterized by the return of naïve B cells (CD19+CD27-IgD+) [31]. This pattern, often called “healthy reconstitution,” indicates that the newly repopulated B cells arise from hematopoietic stem cells in the bone marrow, where they are subjected to strict central tolerance mechanisms [31]. A high naïve-to-memory B cell ratio at 12 months has been linked to lower relapse risk and has been tentatively proposed as a marker of effective immune resetting, though prospective data in pMN remain limited [13]. Beyond descriptive immunophenotyping, genetic evidence further implicates B cell traits in MN susceptibility. A recent bidirectional Mendelian randomization study evaluating 731 immune cell subtypes identified several B cell phenotypes with causal links to pMN, including CD25 expression on IgD+CD24− B cells, which showed reciprocal associations with disease risk [21]. These findings suggest that dysregulation at or near the naïve/early maturation stage may contribute to both the development and persistence of pMN, pointing to the naïve-enriched compartment as a pathogenically relevant target [21].

### 2.3. Class Switched-Memory B Cells (CD19+CD27+IgD-)

Memory B cells are primarily identified by CD27, a TNFR superfamily receptor upregulated after T cell-dependent germinal center reactions, though CD27 can also be acquired through T cell-independent pathways, as in marginal-zone B cells [29]. The memory B cell population is further categorized by the presence or absence of immunoglobulin D (IgD). Cells that have undergone somatic hypermutation and class-switch recombination no longer express IgM or IgD on their surface but instead display IgG, IgA, or IgE.

Class-switched-memory B cells (CD19+CD27+IgD-) are central to pMN pathogenesis. They serve as the immediate, antigen-experienced precursors to short-lived plasmablasts and long-lived plasma cells, the latter representing the main source of sustained high-affinity anti-PLA2R1 IgG4 autoantibody production and driving glomerular injury [19]. Their circulating numbers are not consistently elevated at baseline—in some cohorts they appear low—but this probably reflects preferential homing to secondary lymphoid organs and inflamed renal tissue rather than reduced pathogenic activity [12].

Monitoring the CD19+CD27+IgD- subset is therefore of interest, but the available evidence does not establish it as the unique or the earliest cellular indicator of relapse. In the only cohort to date that has examined relapse prediction systematically in RTX-treated pMN, higher month-6 frequencies of total CD19+, naïve, double-negative (CD27-IgD-) and CD38+ B cells all discriminated relapsers from non-relapsers, with ROC-derived cut-offs of 0.44%, 0.18%, 0.028% and 0.12% respectively [13]. Class-switched memory cells were higher during relapse and reconstituted earlier in relapsers, but did not emerge as an independent early signal superior to the other subsets [13]. Taken together with the data from RTX-treated idiopathic nephrotic syndrome [31], it is more accurate to say that the overall reconstitution pattern, meaning the timing of B cell return and the relative contribution of the naïve, transitional, CD38+, double-negative and memory compartments, carries the prognostic information, and that no single subset has yet been shown to outperform the others in pMN. Whether this sequence is reproducible, and whether it offers sufficient lead time to guide pre-emptive retreatment before recurrent proteinuria develops, has not been tested adequately. This remains a hypothesis rather than an established property of the marker.

### 2.4. Plasmablasts and Long-Lived Plasma Cells (CD19+CD20−IgD−CD27^hi^CD38^hi^/CD138+CD38+CD19−/Low)

Plasmablasts are the most differentiated circulating B cell subset and are directly relevant to understanding why RTX can fail in pMN. Immunophenotypically identified as CD3−CD19+CD20−IgD−CD27^hi^CD38^hi^, they are short-lived, highly active antibody-secreting cells that emerge from germinal center reactions or extrafollicular activation pathways following stimulation of class-switched-memory B cells [19]. Their bone marrow-resident counterparts, long-lived plasma cells (LLPCs), are identified by the phenotype CD138+CD38+CD19−/low and represent the final, fully differentiated effectors of humoral immunity [32]. They can sustain antibody production autonomously for months to years and are the basis of durable humoral memory [32]. The critical shared feature of both subpopulations, directly relevant to RTX therapy, is the absence of CD20 surface expression, which renders them resistant to all anti-CD20 depletion strategies [19]. The two populations, however, differ fundamentally in accessibility: Circulating plasmablasts are quantifiable in peripheral blood by routine flow cytometry and are therefore a candidate biomarker, whereas bone-marrow long-lived plasma cells cannot be assessed without an invasive procedure.

In active, untreated PLA2R1-associated pMN, circulating plasmablasts are elevated relative to healthy controls and correlate closely with anti-PLA2R1 titer levels and immunological disease activity [19]. They are the immediate cellular source of the pathogenic IgG4 autoantibodies that drive subepithelial immune complex deposition and complement-mediated podocyte injury. LLPCs residing in the bone marrow niches and secondary lymphoid organs provide a parallel, sustained source of high-affinity anti-PLA2R1 production that is relatively independent of short-term peripheral CD20+ B cell dynamics [30,32].

Detailed longitudinal monitoring over four years in a single patient with PLA2R1-positive MN treated with RTX demonstrated that, after an initial phase of complete disappearance of CD19+ B cells, plasmablasts and memory B cells by day 15, these cells re-emerged as early as days 45–90, preceding the reappearance of naïve B cells, despite ongoing CD19+ lymphopenia [19]. During follow-up, plasmablast counts remained elevated compared with pre-RTX baseline, and their persistence was accompanied by a progressive rise in anti-PLA2R1 titers, supporting a direct mechanistic link between residual plasmablast activity and ongoing autoantibody production [19]. This kinetic pattern has so far been documented only in a single reported patient, but it points to a likely mechanism of RTX non-response. Even after complete peripheral CD19+ B cell depletion, CD20-negative plasmablasts and LLPCs may continue producing pathogenic anti-PLA2R1 antibodies [12]. In such patients, the presence of persistent or rebounding anti-PLA2R1 titers despite confirmed B cell depletion should therefore raise suspicion of ongoing plasma-cell-driven autoimmunity, alongside other recognized mechanisms of RTX failure such as inadequate drug exposure, extensive baseline epitope spreading, or development of anti-rituximab antibodies [13,19]. This may explain the anti-PLA2R1 titer plateau commonly seen beyond month 3–6 and the suboptimal remission rates with standard 2 × 1g RTX dosing in some patients [13].

### 2.5. Non-Switched-Memory B Cells (CD19+CD27+IgM+IgD+)

The non-switched-memory B cell subset (CD19+CD27+IgM+IgD+) is a unique group of marginal zone-like/unswitched B cells that have acquired memory markers (CD27+) without undergoing isotype class switching, thus retaining surface IgD along with IgM. These cells exhibit phenotypic and functional traits similar to splenic marginal zone B cells [33]. They respond rapidly to T cell-independent antigens, functioning as an innate-like first line of humoral defense.

While the IgG4-driven pathology of pMN is attributed to antigen-specific clones that arise from the switched-memory compartment, non-switched-memory B cells also contribute to the overall inflammatory environment and early immune activation [30]. Baseline assessments in severe pMN have reported decreased circulating levels of these cells, similar to the switched-memory compartment [13]. A longer duration of depletion following RTX therapy is associated with longer clinical remission [31]. Evaluating both switched and non-switched-memory compartments provides a broader picture of the total memory burden; when both remain depleted while naïve cells repopulate, this pattern is consistent with durable immune resetting [31]. However, the strongest prognostic evidence relates to the switched-memory component [19,31].

### 2.6. Regulatory B Cells and Breg-Enriched Phenotypes (CD19+CD5+CD1d^hi^ and CD19+CD24^hi^CD38^hi^)

The expression of the CD5 antigen on B cells has historically been linked to a dualistic functional identity. In certain contexts, CD19+CD5+ cells are classified as “B1-like” innate B cells, known for producing low-affinity antibodies [34,35]. These polyreactive natural autoantibodies can contribute to autoimmune pathology, as seen in conditions like systemic lupus erythematosus (SLE) [34]. In other contexts, particularly when combined with high CD1d expression and IL-10 production, CD19+CD5+CD1d^hi^ cells represent a major regulatory B cell (Breg) subset with immunosuppressive properties [35]. Bregs are functionally defined by their secretion of inhibitory cytokines, principally IL-10 and in some subsets TGF-β and IL-35, which collectively dampen pathogenic immune responses. Via IL-10 and related mediators, Bregs suppress Th1 and Th17 effector cell activation and differentiation, while also promoting Treg expansion and function [35]. Because no lineage-defining transcription factor exists for this compartment, a surface phenotype alone identifies cells enriched for regulatory capacity rather than functional Bregs. We therefore write “Breg-enriched” wherever only surface markers were assessed, and reserve “Breg” for populations in which IL-10 competence was demonstrated, as in the cohort of Ramachandran et al. [20].

In pMN, the available data suggest that the Breg compartment is quantitatively and functionally disturbed. In a cohort of pMN patients treated with cyclical cyclophosphamide and glucocorticoids, the frequency of CD19+CD5+CD1d^hi^ IL-10+ Bregs was significantly lower at baseline than in healthy controls and increased after 8 months of therapy, with greater Breg expansion observed among patients who achieved remission [20]. A second study focusing on the CD19+CD24^hi^CD38^hi^ B cell subset found that these cells were numerically expanded in pMN but displayed a skewed cytokine profile, producing more IL-6 and IL-12 and less IL-10 than in healthy individuals, and their frequency correlated positively with proteinuria and Th17 expansion [23]. Across these studies, Breg-lineage populations appear dysregulated in pMN, either numerically deficient (IL-10-competent CD19+CD5+CD1d^hi^ Bregs) or functionally impaired (CD19+CD24^hi^CD38^hi^ Breg-like cells), thus contributing to a breakdown of peripheral tolerance.

Although detailed longitudinal Breg data in RTX-treated pMN are lacking, B cell–depleting therapy would be expected to deplete Bregs along with other CD19+ subsets, followed by gradual re-emergence during immune reconstitution [31]. Drawing on data from cyclophosphamide-treated pMN [20,23], and from pediatric primary nephrotic syndrome where IL-10-producing Bregs track disease activity and steroid response [36], recovery of functional IL-10-producing Bregs would be expected to accompany clinical remission and lower disease activity. Monitoring CD19+CD5+CD1d^hi^ and related Breg-enriched subsets may therefore reflect a patient’s capacity to restore immune tolerance after B cell depletion. Re-expansion of functional Bregs alongside Treg recovery would represent a favorable immunologic pattern, though it cannot yet be considered a validated disease-specific biomarker. The dynamics of B cell subsets before and after treatment are summarized in Table 3.

## 3. The Lymphocyte Compartment: The Imbalance of Effectors and Regulators

Although pMN is defined by pathogenic autoantibodies, the production of high-affinity, class-switched IgG4 requires specific T cell help [1]. In active pMN, circulating T cells are dysregulated. Chronically activated effector and memory subsets are expanded while regulatory control is substantially diminished [37]. Understanding these T cell dynamics is necessary to interpret RTX response and resistance.

### 3.1. Total T Helper (CD3+CD4+) and Cytotoxic (CD3+CD8+) T Cells

The initiation and maintenance of the autoimmune response in pMN are mainly driven by the CD4+ T helper cell population [38]. On recognizing podocyte autoantigens (such as processed PLA2R1 peptides) presented by MHC class II on B cells or other APCs, naïve CD4+ T cells undergo clonal expansion [38]. These cells subsequently differentiate into specific helper lineages, primarily T follicular helper cells (predominantly Tfh2-skewed in pMN) that provide IL-21 (a pan-Tfh cytokine) together with IL-4, which together support IgG4 class switching [38]. These T follicular helper (Tfh) cells are the key drivers of IgG4 class switching in pMN, supported by CD40-CD40L costimulation [38]. In certain high-risk pMN phenotypes, Th17-mediated inflammation appears more prominent and correlates with higher rates of thromboembolic events and relapse [39].

In untreated pMN, flow cytometry often reveals an abnormally high CD4+/CD8+ ratio in peripheral blood, indicating the systemic expansion of the CD3+CD4+ helper T cell population and the decrease in CD8+ cell counts [22]. However, in the Rosenzwajg 2017 cohort [11] the elevated CD4+/CD8+ ratio was driven primarily by a reduction in CD8+ cell numbers, not by an absolute expansion of CD3+CD4+ T cells; total CD4+ numbers were comparable to controls. After effective RTX treatment, clinical responders show normalization of the CD4+/CD8+ ratio and a significant decrease in circulating CD4+ T cells [22]. This effect is indirect: RTX does not target T cells but, by eliminating CD20+ B cells, it removes a critical source of antigen presentation, depriving autoreactive CD4+ T cells of the stimulation needed to sustain their expanded numbers.

### 3.2. The Naïve and Memory Axis: CD45RA+ Versus CD45RO+ T Cells

Classifying T cell immunological history requires distinguishing antigen-naïve from antigen-experienced memory cells. This is operationally accomplished by analyzing alternative pre-mRNA splicing of the PTPRC gene, which encodes the common leukocyte antigen CD45 [40]. Naïve T cells express the high-molecular-weight isoform, CD45RA+. These cells possess a diverse T cell receptor (TCR) repertoire capable of recognizing a wide range of novel antigens, but they require specific TCR engagement and costimulatory signals (e.g., CD28-B7 interaction) to initiate clonal activation [40]. Following activation and differentiation, T cells switch their pre-mRNA splicing to express the low-molecular-weight isoform CD45RO+. The CD45RA+ isoform retains the exon 4(A), whereas CD45RO+ lacks all three variable exons [40]. The absence of the A, B, and C exons in the CD45RO isoform modifies its extracellular domain, enabling closer physical contact with the T cell receptor complex and its associated kinases (such as Lck) [40,41]. The compact extracellular domain of CD45RO (lacking all three exons) prevents its steric exclusion from the immunological synapse, allowing it to more readily dephosphorylate the inhibitory phosphorylation site (Tyr505) of Lck, thereby facilitating more efficient TCR signaling and contributing to the lower activation threshold of memory T cells [41].

In active, severe pMN, the total T cell pool is pathologically skewed toward the CD45RO+ memory phenotype, reflecting systemic accumulation of long-lived, antigen-experienced clones [11]. This shift is concentrated in the helper compartment where both the percentage and absolute number of CD45RO+ memory T helper cells are elevated. In contrast, the CD45RA+ naïve T helper cell pool is reduced [11]. This expanded CD45RO+ population signifies the formation of a strong autoimmune memory pool directed against podocyte antigens. These cells home preferentially to inflamed tissues and continuously support B cell activation and autoantibody production [39].

The longitudinal monitoring of the CD45RA/CD45RO axis offers valuable prognostic insights. In active pMN, both the percentage and absolute number of CD4+CD45RO+ effector and central memory T cells are significantly elevated compared with healthy controls, and their levels have been found to be associated with disease activity [38]. Following RTX therapy, clinical responders have been reported to show a decrease in circulating CD45RO+ central and effector memory T cells [22]. This shift suggests that chronic autoimmune activation has been suppressed. Mechanistically, the presence of persistently elevated CD45RO+ effector memory T cells despite adequate B cell depletion may signal an impending relapse. In this setting, the autoreactive T cell memory compartment retains the capacity to rapidly reactivate B cells during reconstitution. However, this hypothesis requires prospective validation in dedicated longitudinal pMN cohorts.

### 3.3. Regulatory T Cells (Tregs) (CD4+CD25+FoxP3+)

Regulatory T cells (Tregs) are a specialized CD4+ T cell subset central to the maintenance of peripheral immune tolerance. They are characterized immunophenotypically by a constitutively high expression of the interleukin-2 receptor alpha chain (CD25) and, most notably, by the intracellular presence of the key transcription factor Forkhead box P3 (FoxP3) [42]. FoxP3 is critical for the development and suppressive function of Tregs. Tregs help maintain immune system balance by actively inhibiting the proliferation and cytokine secretion of autoreactive CD4+ and CD8+ effector T cells, as well as indirectly preventing pathogenic B cell activation and class switching by restraining CD4+ T helper cell activity [43]. These effects are mediated by inhibitory cytokines (TGF-β, IL-10, and IL-35) and CTLA-4-dependent APC modulation [43].

In active pMN, circulating Tregs are consistently reduced, often with lower FOXP3 and TGF-β expression, indicating impaired regulatory function [34]. The resulting loss of regulatory control permits unchecked expansion of CD4+CD45RO+ memory helper cells and excessive activation of autoantibody-producing B cells [20,43].

Baseline circulating Treg levels have been proposed as a candidate marker of early RTX response, although the supporting evidence rests essentially on a single study. In the cohort reported by Rosenzwajg et al., 16 patients with severe MN who subsequently achieved clinical remission had significantly lower baseline Treg percentages than non-responders, and showed a rapid increase in circulating Tregs detectable as early as day 8 after infusion, whereas non-responders showed persistently low Tregs and no post-treatment expansion [11]. This pattern is biologically coherent and, if confirmed, would be attractive because it is detectable within days of the first infusion. It should nevertheless be regarded as a candidate response marker rather than a validated predictor. This pattern derives from a small single-center cohort, in which the reported results are percentages, unaccompanied by absolute counts. It is unknown whether an early Treg rise predicts durable long-term tolerance or merely short-term response. The dynamics of T cell subsets before and after treatment are summarized in Table 4.

## 4. Monocyte Heterogeneity: Innate Drivers of Glomerular Inflammation and Fibrosis

Monocytes bridge innate and adaptive immunity, connecting early nonspecific inflammation to downstream adaptive immune activation. In pMN, circulating monocytes and their tissue derivatives appear to amplify immune complex-mediated glomerular injury and contribute to tubulointerstitial fibrosis [16,44].

Human monocytes are categorized into three phenotypically and functionally distinct subsets based on the relative surface expression of the lipopolysaccharide (LPS) coreceptor CD14 and the low-affinity Fc-gamma receptor III CD16: classical (CD14++CD16-), intermediate (CD14++CD16+), and non-classical (CD14+CD16++) monocytes [45]. Each subset has been implicated in different aspects of systemic inflammation, vascular injury, and CKD [46,47,48].

### 4.1. Classical Monocytes (CD14++CD16-)

Classical monocytes (CD14++CD16-) constitute approximately 80% to 90% of the circulating monocytes in healthy individuals [45]. They are highly phagocytic cells that primarily clear pathogens, cellular debris, and apoptotic bodies. They express high levels of chemokine receptors, such as CCR2, enabling recruitment to sites of acute tissue injury [45]. Classical monocytes drive acute inflammation, but in chronic inflammatory and fibrotic conditions, CD16+ subsets, both intermediate and non-classical, are more consistently implicated in sustained antigen presentation, endothelial injury, and atherogenesis [49]. In CKD and coronary artery disease, higher proportions of intermediate monocytes and a relative decrease in classical monocytes have been found to be associated with increased risk of major adverse cardiovascular events [50].

### 4.2. Intermediate Monocytes (CD14++CD16+)

Intermediate monocytes (CD14++CD16+) form a highly pro-inflammatory, metabolically active subset that occupies a transitional position in the monocyte lineage [45]. These cells are uniquely identified by their exceptionally high surface expression of major histocompatibility complex (MHC) class II molecules, rendering them powerful professional antigen-presenting cells capable of engaging and activating CD4+ T cells [51]. When stimulated, intermediate monocytes release large amounts of pro-inflammatory cytokines, including tumor necrosis factor-alpha (TNF-α), interleukin-1 beta (IL-1β), and interleukin-6 (IL-6) [52].

In IgA nephropathy and advanced CKD, intermediate monocytes are expanded in peripheral blood and correlate with markers of systemic inflammation and endothelial dysfunction [53,54]. Comparable pMN data are beginning to emerge; transcriptomic profiling of peripheral blood mononuclear cells from newly diagnosed pMN patients reveals a strongly pro-inflammatory monocyte signature (IL1B, IL15, CXCL8, CXCL10, NLRP3, cGAS-IFI16), consistent with CD16+ subset activation or expansion, though precise changes in intermediate monocyte frequency have not been quantified [55].

### 4.3. Non-Classical Monocytes (CD14+CD16++)

Non-classical, or “patrolling,” monocytes (CD14+CD16++) represent a highly mature subset primarily involved in monitoring the vascular endothelium for damage, resolving acute inflammation, and supporting subsequent tissue remodeling [56]. They represent the terminal stage of the accepted ontogenetic sequence, arising from the intermediate compartment through progressive downregulation of CD14 [45]. No longitudinal data are available for this subset in pMN, and its behavior under anti-CD20 therapy has not been reported. It is presented here for completeness of the CD14/CD16 classification rather than as a candidate biomarker, and the inferences that can be drawn about CD16-expressing monocytes in glomerular disease rest predominantly on CKD and IgA nephropathy cohorts.

### 4.4. M2-like Monocytes (CD14+CD163+CD206+)

The phenotypes discussed in this subsection belong to a different classification system from those in Section 4.1, Section 4.2 and Section 4.3, and the two should not be used interchangeably. The CD14/CD16 scheme defines three ontogenetically related circulating subsets, classical, intermediate and non-classical, and is a nomenclature for cells in peripheral blood [45]. The M1/M2 scheme describes polarized activation states, derived largely from in vitro macrophage stimulation, and is applied to circulating monocytes only by analogy, through surrogate markers such as CD163 and CD206 [16]. A CD14+CD163+CD206+ “M2-like” monocyte is therefore not equivalent to a non-classical CD14+CD16++ monocyte. The two are defined by different antibody panels, and the populations overlap only partially. Where studies are compared below, the classification used in the original report is retained.

In early pMN, circulating monocytes with an M2-like phenotype (CD14+CD163+CD206+) are expanded, and their numbers correlate with the degree of proteinuria and PLA2R1 antibody titers [16]. These M2-biased cells overlap phenotypically with non-classical and CD16+ monocyte subsets but are defined more by their scavenger-receptor expression and cytokine profile than by CD16 alone [16]. The physiological role of M2-like monocytes is to reduce inflammation and coordinate the repair of damaged extracellular matrix [16]. In pMN, however, this repair function becomes pathological. While M2-like monocytes and macrophages initially limit acute inflammation and support matrix repair, sustained activation in MN is linked to upregulation of profibrotic mediators including TGF-β, and to progressive glomerulosclerosis and tubulointerstitial fibrosis [16].

In a small cohort of patients with early pMN treated with tacrolimus plus low-dose prednisone for 12 weeks, proteinuria decreased, serum albumin increased, but circulating CD14+CD163+CD206+ M2-like monocytes did not significantly decline compared with baseline and remained significantly higher than in healthy controls [16]. These findings suggest that M2-like monocyte-driven matrix remodeling and profibrotic signaling may persist even after partial clinical remission, though the sample was small and replication in larger RTX-treated pMN cohorts is needed.

### 4.5. Monocyte Activation and the Systemic Inflammation Response Index (SIRI)

Beyond subset frequency, the functional activation state of the total monocyte compartment is assessed by evaluating the surface expression of the human leukocyte antigen–DR isotype (HLA-DR). The expression of CD14++HLA-DR+ identifies a population of highly activated monocytes capable of strong antigen presentation to the adaptive immune system [51]. Mendelian randomization studies suggest that genetically higher HLA-DR expression on CD14+ and CD14+CD16− monocytes increases the risk of progressive kidney diseases such as diabetic nephropathy, supporting a pathogenic role for sustained monocyte antigen-presenting activation in CKD [57]. Whether similar genetic relationships exist in pMN has not yet been demonstrated. In contrast, very low HLA-DR expression marks systemic immune paralysis in sepsis [58]. How longitudinal changes in monocyte HLA-DR expression during RTX therapy relate to clinical response in pMN has not been systematically studied, and warrant prospective validation.

The clinical value of monocyte monitoring increases when these measures are combined into composite indices such as the Systemic Inflammation Response Index (SIRI), which incorporates peripheral monocytes, neutrophils, and lymphocyte counts [18]. A recent single-center cohort examining pMN patients treated with RTX demonstrated that non-responders consistently have high SIRI scores throughout the 12-month observation period [15].

Combining SIRI with B cell depletion metrics improves prognostic accuracy. At month 3 post-RTX, a low SIRI score (≤1.25) combined with significant peripheral B cell depletion (≤0.2%) is a strong independent predictor of clinical remission by month 6 [15]. Adding these two cellular measures to traditional predictive models—which rely solely on proteinuria, albumin, and anti-PLA2R1 antibodies—significantly improves the area under the curve (AUC) from 0.81 to 0.86 [15]. This cohort, however, reports percentages only and no absolute counts. By month 6, the extent of B cell depletion and anti-PLA2R1 antibody levels often no longer predict outcome, because most patients have achieved depletion by that point. At this point, only a SIRI score ≤0.9 and the incremental rise in serum albumin remain as independent predictors of a long-term (12-month) prognosis [15]. Once humoral B cell activity is suppressed, the residual driver of long-term renal outcomes appears to be the innate, monocyte-driven inflammation [15,17]. Unlike the subsets discussed above, the systemic inflammation response index is derived entirely from the routine differential white cell count and requires no flow cytometry, no dedicated antibody panel and no specialized laboratory infrastructure. It is therefore considered here as an inflammatory prognostic index rather than as an immunophenotypic biomarker, and its low cost and universal availability make it the parameter most readily transferable to routine practice, notwithstanding its lack of cellular specificity. The dynamics of the monocyte compartment before and after treatment are summarized in Table 5.

## 5. Mechanisms of RTX-Mediated Immune Network Modulation

RTX works in pMN primarily by eliminating CD20+ B cells, the cellular source of pathogenic anti-PLA2R1 autoantibodies (70–80% of cases) [8]. Immunophenotypic profiling, however, has made it clear that RTX does more than just deplete B cells; it also alters the broader immune compartment, with significant secondary effects on T cell subsets and monocyte populations [11,40,59]. A deeper understanding of these mechanisms is needed to accurately interpret cellular biomarkers.

### 5.1. Primary Mechanisms of B Cell Clearance and Pharmacokinetic Challenges

RTX is a chimeric, type I monoclonal IgG1 antibody that binds specifically to the CD20 antigen [9]. CD20 is a transmembrane phosphoprotein expressed on cells of the B lymphocyte lineage from the late pre-B cell stage through to mature and memory B cells [60]. Crucially, CD20 is absent on hematopoietic stem cells, pro-B cells, and terminally differentiated, long-lived plasma cells [61]. Upon binding to CD20, RTX mediates the rapid depletion of peripheral B cells through three principal mechanisms, namely complement-dependent cytotoxicity (CDC), achieved by recruiting the C1q and downstream complement components; antibody-dependent cellular cytotoxicity (ADCC), primarily executed by NK cells; and antibody-dependent cellular phagocytosis (ADCP), executed by tissue macrophages, with CDC predominating in vitro and ADCP considered the main effector mechanism in vivo for Type I anti-CD20 antibodies [62].

**Figure 1:**
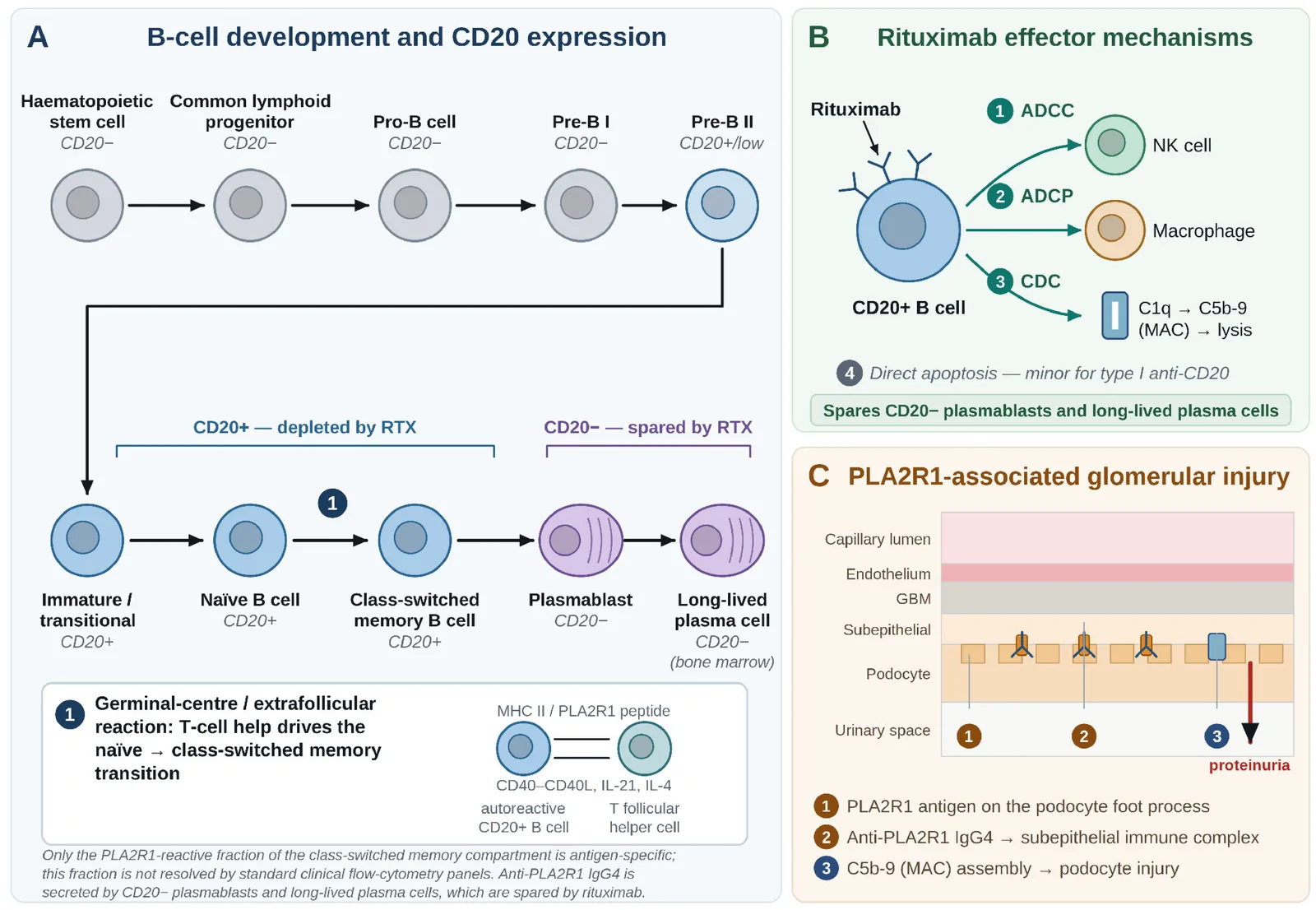
B cell development, rituximab effector mechanisms and PLA2R1-associated glomerular injury in pMN. (**A**) B-cell development and CD20 expression. CD20 is acquired at the pre-B II stage and retained through the immature/transitional, naïve and class-switched memory stages. Expression is lost on terminal differentiation, making plasmablasts and bone-marrow long-lived plasma cells CD20-negative and therefore not depleted by anti-CD20 therapy. The inset shows the germinal-centre/extrafollicular reaction in which T-cell help drives the naïve → class-switched memory transition. (**B**) Rituximab effector mechanisms. Rituximab bound to CD20 engages antibody-dependent cellular cytotoxicity via NK cells, antibody-dependent cellular phagocytosis via macrophages, and complement-dependent cytotoxicity through C1q and C5b-9. Direct apoptosis is a minor contributor for type I anti-CD20 antibodies. (**C**) PLA2R1-associated glomerular injury. Anti-PLA2R1 IgG4 binds PLA2R1 on the podocyte foot process, forming subepithelial immune complexes. C5b-9 assembly causes podocyte injury and proteinuria. Abbreviations: pMN: primary membranous nephropathy, RTX: rituximab, HSC: hematopoietic stem cell, PLA2R1: M-type phospholipase A2 receptor 1, MHC II: major histocompatibility complex class II, Tfh: T follicular helper cell, IL: interleukin, ADCC: antibody-dependent cellular cytotoxicity, ADCP: antibody-dependent cellular phagocytosis, CDC: complement-dependent cytotoxicity, MAC: membrane attack complex, NK: natural killer cell, GBM: glomerular basement membrane.

In patients with nephrotic-range, non-selective proteinuria, case reports and PK studies have shown profound alterations in RTX disposition. In particular, RTX serum half-life can drop from approximately 20 days in non-nephrotic patients to as low as <1 day in severe nephrotic syndrome, with urinary clearance accounting for at least 25% of total drug elimination in extreme cases compared with ~0% in healthy controls [63]. In larger pMN cohorts, population PK models still demonstrate shortened half-life and increased clearance relative to non-nephrotic diseases [64]. Population PK analyses suggest that baseline proteinuria explains a substantial fraction of inter-individual variability in RTX clearance [65]. These PK alterations substantially reduce RTX bioavailability compared with non-nephrotic conditions such as rheumatoid arthritis or B cell lymphoma. Given these pharmacokinetic constraints, flow-cytometric assessment of CD19+ B cells early after infusion (e.g., day 15 or 1 month) is the only practical means of confirming adequate target engagement before concluding that true biological resistance is present. The B-cell development, rituximab effector mechanisms and PLA2R1-associated glomerular injury in pMN are presented in Figure 1.

### 5.2. A Hypothesized NK Cell–TGF-β–Treg Axis

One notable aspect of RTX’s mechanism in pMN is its indirect effect on the CD4+ T cell compartment [11,22]. Although CD4+CD25+FOXP3+ regulatory T cells (Tregs) do not express CD20, several studies have shown that they are significantly modulated by RTX therapy in pMN, with early post-treatment Treg expansion associating with subsequent clinical remission [11]. During ADCC, NK cells recognize RTX-opsonized B cells via the FcγRIIIa (CD16a) receptor, leading to immune synapse formation, degranulation with perforin and granzymes, and B cell lysis [66,67]. Strong CD16a engagement triggers proteolytic shedding of surface CD16a, concurrent with NK-cell degranulation and cytokine release (e.g., IFN-γ, TNF-α) [66,67]. In the broader immune microenvironment, TGF-β together with IL-2 can drive peripheral conversion of naïve CD4+ T cells into induced Tregs via FOXP3 induction, with TGF-β providing the lineage-specifying signal and IL-2/STAT5 signaling stabilizing FOXP3 expression [68,69].

Early post-RTX rises in circulating Tregs have been reported as a cellular biomarker of favorable outcome in pMN [11]. A plausible interpretation is that RTX-opsonized B cells activate NK cells through FcγRIIIa, and that the resulting TGF-β- and IL-2-rich environment then supports peripheral FOXP3+ Treg induction or expansion. Obinutuzumab, a glycoengineered, afucosylated type II anti-CD20 antibody with enhanced FcγRIIIa affinity and ADCC potency [70], fits this same mechanistic scheme. Enhanced FcγRIIIa ligation by obinutuzumab leads to faster, more profound CD16a down-modulation and stronger NK-cell activation in vitro, resulting in superior ADCC compared with RTX [70,71]. Obinutuzumab has induced high remission rates in several cohorts with RTX-refractory MN [72,73]. More recently, the phase 3 MAJESTY trial provided randomized evidence in treatment-naïve pMN, with obinutuzumab achieving a markedly higher complete remission rate at week 104 than tacrolimus (37% vs. 6%) and substantially fewer relapses after remission, and no new safety signals [10]. These features suggest obinutuzumab may more effectively engage the NK–TGF-β–Treg pathway in RTX-refractory pMN, though direct Treg dynamics data under obinutuzumab are still lacking. However, no study in pMN has measured NK-cell activation, TGF-β and IL-2 availability, and Treg induction in the same patients. Each individual step is supported by work in other systems, and the pathway as a whole is therefore advanced as a mechanistic hypothesis to account for the reproducible clinical observation of early post-RTX Treg expansion, not as an established mechanism.

### 5.3. Downstream Alterations of Effector T Cells and Monocytes

**Figure 2:**
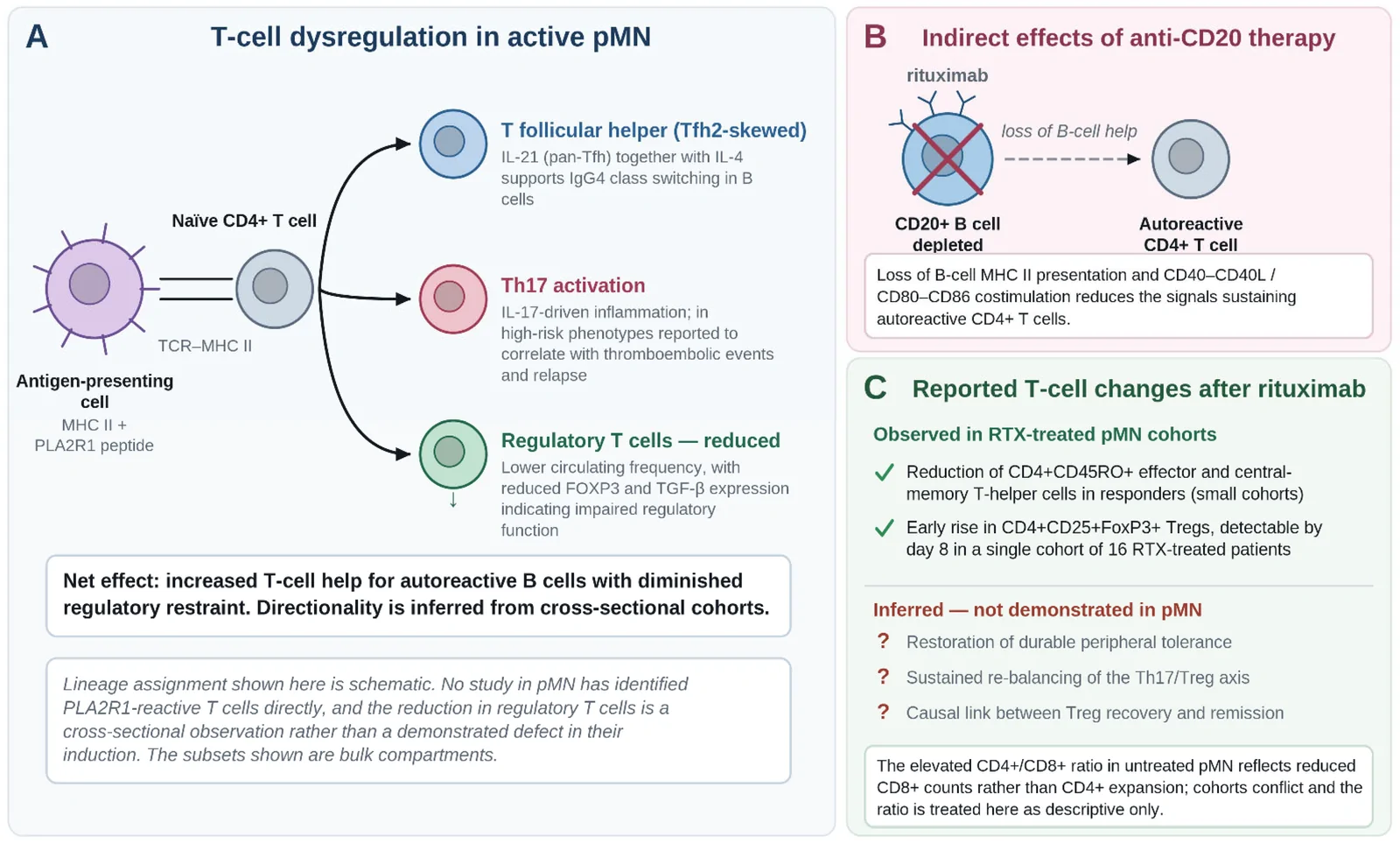
T cell dysregulation in active pMN and reported T cell changes during anti-CD20 therapy. (**A**) T-cell dysregulation in active pMN. Antigen-presenting cells display processed PLA2R1 peptide on MHC class II to naïve CD4+ T cells, which differentiate into Tfh2-skewed, Th17 and regulatory lineages. The regulatory compartment is reduced in active disease. (**B**) Indirect effects of anti-CD20 therapy. Depletion of CD20+ B cells removes B-cell antigen presentation and costimulation to autoreactive CD4+ T cells. (**C**) Reported T-cell changes after rituximab, with changes observed in RTX-treated pMN cohorts separated from inferences that have not been demonstrated in pMN. Abbreviations: pMN: primary membranous nephropathy, RTX: rituximab, PLA2R1: M-type phospholipase A2 receptor 1, MHC II: major histocompatibility complex class II, TCR: T cell receptor, Tfh: T follicular helper cell, Th17: T helper 17 cell, Treg: regulatory T cell, FOXP3: Forkhead box P3, TGF-β: transforming growth factor beta, IL: interleukin.

Anti-CD20-mediated depletion of CD19^+^ B cells substantially reduces the antigen presentation and costimulatory signals that CD4^+^ T cells require to sustain chronic autoreactive activity [74,75]. Without ongoing B cell-driven MHC class II presentation and CD40–CD40L/CD80–CD86 costimulation, differentiated CD4^+^ memory T-helper cells gradually decline in frequency, and naïve and central-memory subsets can partially re-expand, as observed in anti-CD20-treated lymphoma and idiopathic nephrotic syndrome cohorts [31,76]. T-cell dysregulation in active pMN and post-RTX effect in T-cells is presented in Figure 2.

In parallel, RTX-induced clearance of subepithelial immune complexes and dampening of T-helper-dependent effector pathways reduce intrarenal complement activation and chemokine production, thereby diminishing key chemotactic signals, such as MCP-1/CCL2, that drive monocyte recruitment and activation in glomerular disease [77,78]. Although pMN is characterized by the expansion of circulating CD14^+^CD163^+^CD206^+^ M2-like monocytes rather than specifically defined CD14++CD16^+^ intermediate subsets, the overall reduction in T cell-dependent inflammation and immune complex burden would be expected to attenuate the pathological activation and trafficking of pro-inflammatory monocyte/macrophage populations [16]. These effects of anti-CD20 therapy extend well beyond B cell depletion, with downstream effects on both adaptive and innate immunity, reducing systemic inflammatory activity.

## 6. Toward a Biomarker-Informed Framework: Hypotheses for Future Validation

The 2021 KDIGO Clinical Practice Guidelines recommend personalized management of pMN based on risk stratification, currently anchored on clinical parameters (proteinuria, eGFR) and serology (anti-PLA2R1 titers) [79,80]. The cellular observations summarized in this review raise the possibility that immunophenotyping could complement this framework, though the evidence base supporting any specific application remains preliminary. Most published cellular biomarker data in pMN derive from small, single-center, non-randomized cohorts, and several inferences in the discussion below rely on extrapolation from related antibody-mediated diseases treated with RTX, including idiopathic nephrotic syndrome (INS), ANCA-associated vasculitis, lupus nephritis, and rheumatoid arthritis. There is no validated panel, no validated cut-off, no evidence that any cellular measurement adds to proteinuria, eGFR and anti-PLA2R1 titer in a way that changes management, and no trial in which a cellular measurement has been used to direct therapy. None of the proposals that follow have been validated in a prospective randomized MN trial. The framework below is therefore offered as a structured set of hypotheses for future validation, not as an actionable treatment algorithm.

### 6.1. Baseline Profiling: Candidate Predictors of Response and Resistance

Before the first RTX dose, cellular profiling could in principle help stratify patients by response likelihood, but every candidate marker discussed below requires prospective validation in an adequately powered MN cohort before clinical use can be recommended.

- Regulatory T Cell Profiling: In a single mechanistic study, low baseline Treg percentage was associated with impaired regulatory function, and RTX-treated patients in this subgroup showed early Treg expansion and a more favorable clinical course [11].
- Innate Monocyte Indices: Elevated CD14+CD163+CD206+ M2-like monocytes and high baseline SIRI scores have been correlated with greater systemic inflammation and proteinuria in observational MN cohorts [15,16]. It has been hypothesized that highly inflamed patients may experience reduced effective RTX exposure, possibly through greater urinary antibody loss; whether this translates into a need for adjusted dosing has not been formally tested in MN, and any such strategy would require prospective evaluation before being adopted in practice.

### 6.2. Month 3: Evaluating the Depletion Nadir and Early Response

The three-month time point appears to offer the earliest informative window on pharmacodynamic efficacy. Clinically meaningful proteinuria reduction often lags behind, due to the slow clearance of existing subepithelial deposits and the time required for podocyte recovery.

- Total and Switched-memory B cell Eradication: In retrospective and observational data, failure to achieve CD19+ counts < 5 cells/µL or <1% at three months has been interpreted as suboptimal depletion, prompting evaluation for underdosing, increased drug clearance, or anti-rituximab antibodies (ARAs), particularly when accompanied by rapid B cell reconstitution [27,81]. The threshold for “adequate” depletion in MN specifically is not well established and is largely extrapolated from rheumatologic and pediatric nephrology cohorts. The possibility that near-complete depletion of CD19+CD27+IgD− switched-memory B cells identifies a lower-risk subgroup is currently supported indirectly by INS and other autoimmune studies [31,82]. Direct MN evidence is limited.
- Monocyte and Inflammatory Monitoring: In one single-center cohort of 149 patients, an early reduction in the SIRI score (≤1.25) at month 3 combined with B cell depletion ≤ 0.2% was associated with a higher probability of clinical remission by month 6 [15]. This finding, while encouraging, has not yet been independently replicated, and its operating characteristics in geographically and demographically distinct populations are unknown.

### 6.3. Months 6 to 12: Assessing Reconstitution and Tolerance Maintenance

During the therapy maintenance phase, the clinical focus shifts from monitoring depletion kinetics to assessing the quality of immunological reconstitution. This period is crucial in determining whether the patient will sustain long-term remission or eventually relapse.

**Figure 3:**
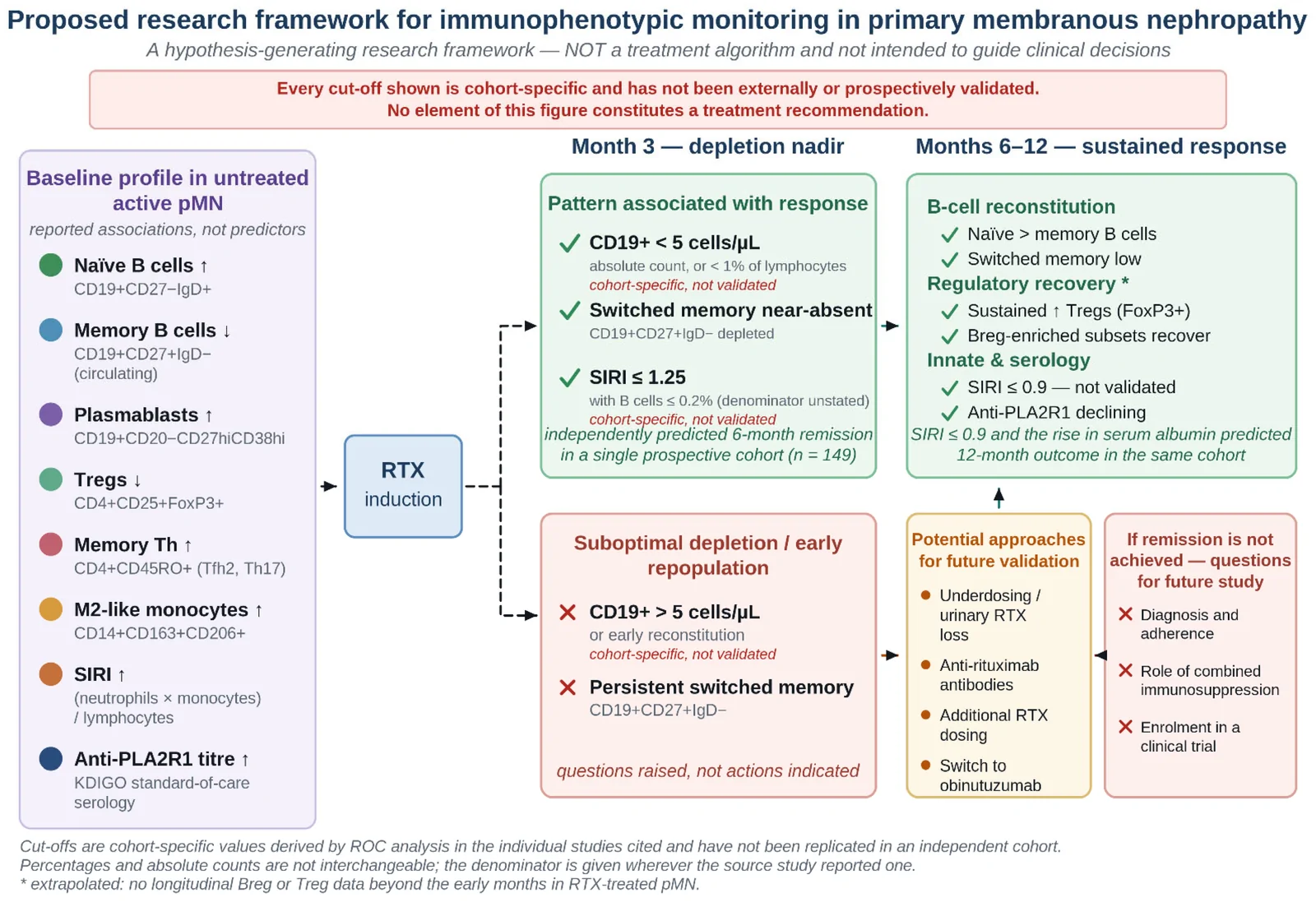
Proposed research framework for immunophenotypic monitoring in RTX-treated pMN. Note: This figure presents a hypothesis-generating research framework. It is not a treatment algorithm and no element of it constitutes a clinical recommendation. All cut-offs shown are cohort-specific values derived by ROC analysis within the individual studies cited and have not been replicated in an independent cohort or validated prospectively. The asterisk denotes extrapolation: no longitudinal Breg or Treg data beyond early months exist in RTX-treated pMN. Abbreviations: pMN: primary membranous nephropathy, RTX: rituximab, KDIGO: Kidney Disease: Improving Global Outcomes, SIRI: Systemic Inflammation Response Index, PLA2R1: M-type phospholipase A2 receptor 1, Treg: regulatory T cell, Breg: regulatory B cell, Tfh: T follicular helper cell, Th17: T helper 17 cell, ROC: receiver operating characteristic.

- The B Cell Reconstitution Pattern: A naïve-predominant return of B cells, rather than early re-expansion of memory subsets, has been associated with lower relapse risk in pediatric INS and other RTX-treated nephrotic syndromes [31,82].
- Restoration of Regulatory Subsets: A sustained increase in CD4+CD25+FoxP3+ Tregs, and possibly in CD19+CD5+ (or other Breg-enriched) subsets compared with baseline, suggests re-establishment of a more tolerogenic immune environment, although Breg data in RTX-treated pMN are still limited [11,20].
- Resolution of Innate Inflammation: In the Duan 2026 cohort, a SIRI value above 0.9 at months 6–12 was associated with a markedly lower probability of remission at 12 months and remained an independent adverse prognostic factor after adjustment for conventional markers [15]. Independent confirmation of this threshold and of its prognostic independence is needed. A proposed research framework for immunophenotypic monitoring in RTX-treated pMN is presented in Figure 3.

### 6.4. Cellular Signatures of Relapse: Hypothesis-Generating Observations

Clinical relapse in pMN is typically heralded by rising anti-PLA2R1 titers, which themselves usually precede proteinuria. Whether cellular changes can provide an even earlier signal is biologically plausible but still unproven.

**Figure 4:**
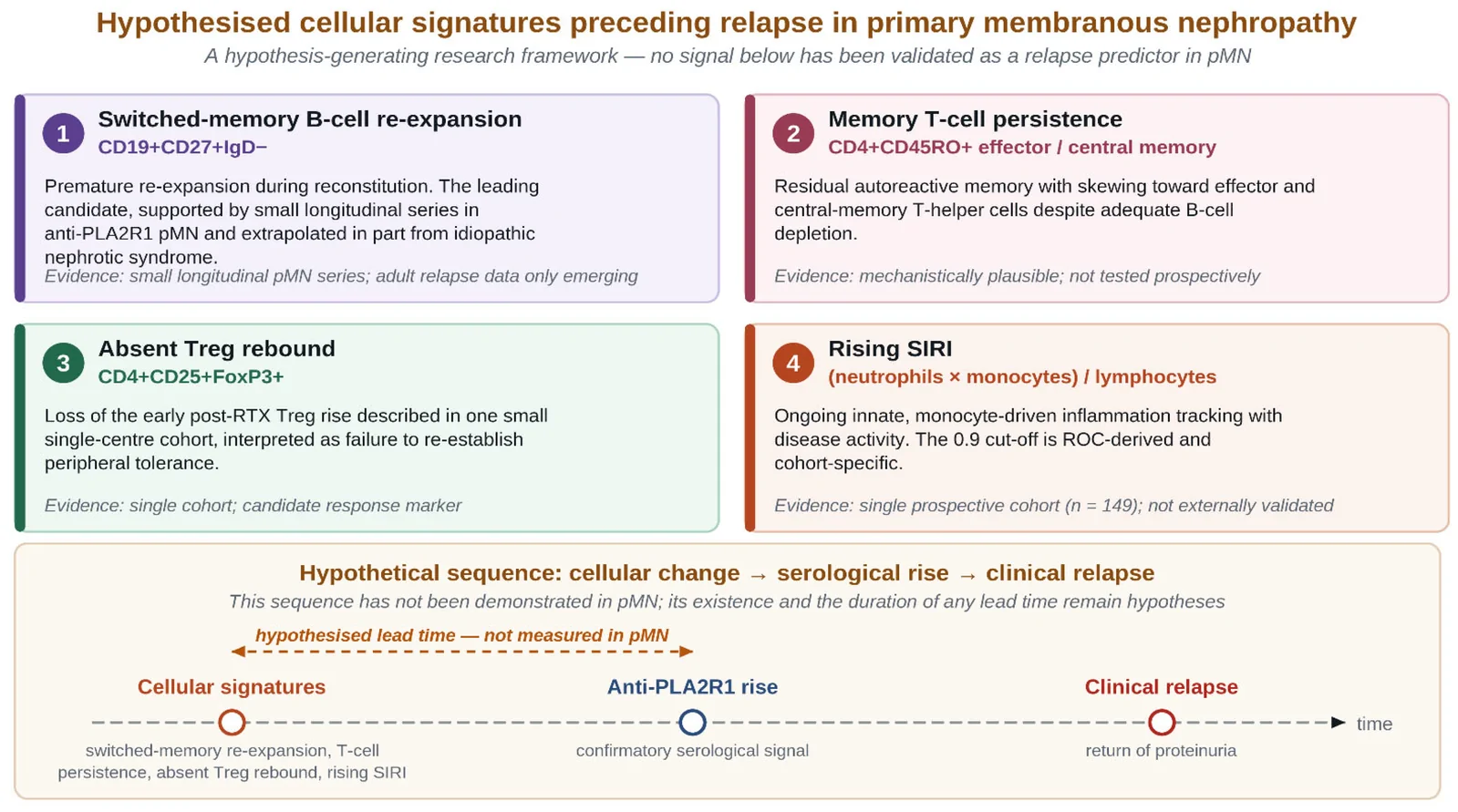
Hypothesized cellular signatures preceding relapse in pMN. Note: This figure presents a hypothesis-generating research framework and does not constitute a clinical recommendation. None of the four signatures has been validated as a relapse predictor in pMN. The temporal sequence in the lower panel is hypothetical and has not been demonstrated in pMN. The SIRI cut-off shown is ROC-derived and cohort-specific. Abbreviations: pMN: primary membranous nephropathy, RTX: rituximab, SIRI: Systemic Inflammation Response Index, PLA2R1: M-type phospholipase A2 receptor 1, Treg: regulatory T cell, ROC: receiver operating characteristic.

- Reconstitution profile at month 6: A reconstitution pattern characterized by higher total CD19+, naïve double-negative and CD38+ B cell frequencies has been associated with subsequent relapse in one prospective RTX-treated pMN cohort, and premature expansion of switched-memory cells has been proposed as an early indicator by extrapolation from idiopathic nephrotic syndrome and other RTX-treated autoimmune diseases. These cut-offs are cohort-specific, derived by ROC analysis without external validation, and require prospective confirmation before any of them can be used to identify re-emergence of autoreactive clones [13,19,31,82,83].
- Memory T Cell Persistence: Persistent skewing toward CD4^+^CD45RO^+^ memory Th cells could theoretically suggest residual effector memory activity that could facilitate rapid restimulation of newly reconstituted B cells; this is mechanistically plausible but remains to be defined prospectively as a relapse biomarker in MN [11,38]. The hypothesized cellular signatures preceding relapse in pMN, are presented in Figure 4.

### 6.5. Limitations of the Study

Several limitations constrain the conclusions drawn above. First, with the exception of one prospective cohort of 149 RTX-treated patients [15], the cellular data in pMN come from single-center studies of a few dozen patients. None of the studies randomized patients to a biomarker-guided strategy, and most studies report associations rather than predictive performance. Second, the studies cited used different antibody panels, fluorochromes and gating strategies, and different operational definitions of the same nominal subset, making reported frequencies not directly comparable across studies. Results are further reported as percentages in some cohorts and as absolute counts in others, frequently without stating the denominator, and sample handling differs as well: Results obtained from fresh whole blood and from cryopreserved peripheral blood mononuclear cells are not directly comparable, which is rarely stated.

Third, treatment regimens varied between studies. RTX regimens differed substantially between cohorts (two 1 g infusions two weeks apart; 375 mg/m^2^ weekly for four weeks; single-dose and weight-adjusted schedules; with and without maintenance), and pharmacokinetic exposure differs further in nephrotic patients because of urinary drug loss. Concomitant immunosuppression, intercurrent infection, and the lymphopenia and hemodilution that accompany the nephrotic state all alter circulating subset distributions independently of the underlying autoimmune process, and are seldom reported or adjusted for. Fourth, measurements were made at inconsistent intervals, and timing relative to infusion largely determines whether a subset appears depleted, reconstituting or recovered. Conflicting results may reflect sampling schedules rather than biology. Fifth, several proposed relapse and reconstitution signatures rest principally on data from idiopathic nephrotic syndrome, ANCA-associated vasculitis, lupus nephritis or rheumatoid arthritis. Although all are B cell-dependent diseases, differences in target antigen, dominant IgG subclass and site of injury mean that transfer to pMN cannot be assumed. Lastly, almost all longitudinal cellular data in pMN derive from anti-PLA2R1-positive disease. The Duan et al. cohort required an anti-PLA2R1 titer of at least 2 RU/mL for inclusion, the relapse-prediction cohort of Cheddadi et al. enrolled PLA2R1-positive patients only, and the remaining mechanistic cohorts were likewise dominated by PLA2R1-associated disease. No published study has profiled peripheral immune cell subsets longitudinally in pMN associated with other antigens, or in antigen-negative disease. Prospective validation of any of the parameters discussed will therefore require harmonized panels, a pre-specified gating strategy applied centrally, standardized sampling time points relative to infusion, and reporting of both percentages and absolute counts. For these reasons the framework in Section 6 and the signatures in Figure 3 and Figure 4 are offered as hypotheses to be tested, not as recommendations.

## 7. Conclusions

In pMN, CD27+IgD− switched-memory B cells and CD45RO+ memory T-helper cells drive the antigen-specific response against PLA2R1 (and, less commonly, THSD7A), while pro-inflammatory and profibrotic monocyte subsets sustain glomerular injury; the regulatory compartments that would normally restrain this response, specifically, CD4+CD25+FoxP3+ Tregs and CD19+CD5+ Bregs, are depleted and functionally impaired, leaving the autoimmune loop largely unopposed. RTX acts on this system at several levels, depleting CD20+ B cells, lowering anti-PLA2R1 production, engaging NK-cell effector pathways, and, within a TGF-β/IL-2-rich milieu, potentially favoring Treg expansion [10,68]. Positioned as an adjunct to the KDIGO framework, immunophenotypic profiling could in principle help stratify baseline risk, capture early pharmacodynamic responses to RTX, and signal the re-emergence of autoreactive clones before clinical or serological relapse.

These inferences, however, derive largely from small, single-center cohorts using heterogeneous flow-cytometry panels, and many proposed relapse and reconstitution signatures are extrapolated from related B cell-driven diseases rather than validated in pMN; they should be regarded as hypothesis-generating. Whether biomarker-guided dosing improves hard renal outcomes remains untested. Prospective multicenter validation of standardized panels and candidate cut-offs—together with comparative profiling in PLA2R1-positive versus PLA2R1-negative disease and integration into multivariate models alongside serological and histological data—is the priority before immunophenotyping can move from a mechanistically informative tool to a clinically actionable one. On the available evidence, no cytometry-based cell profiling can currently be recommended for therapy monitoring in pMN.

Specifically, the necessary next step is a prospective multicenter cohort of incident pMN patients treated with RTX, using a harmonized flow-cytometry panel with centrally standardized gating, pre-specified candidate cut-offs, and sampling at baseline, 3, 6, 12 and 24 months, powered for the KDIGO-defined remission endpoint at 24 months and analyzed alongside anti-PLA2R1 titers in multivariable models.

## Figures and Tables

**Table 1:** Studies assessing adaptive and innate immune cell subsets and composite inflammatory indices (NLR, MLR, SIRI) in pMN.

Author/Year	Subjects	Immune Cell Subset	Findings	Notes
Cantarelli et al., 2020 [12]	pMN patients (*n* = 30), non-immune-mediated CKD patients (*n* = 31; diabetic nephropathy *n* = 9, hypertensive nephropathy *n* = 8, ADPKD *n* = 5, unspecified CKD *n* = 9), and HCs (*n* = 12). No treatment assigned, only baseline assessment was measured	Plasma cells, B cells (Breg, naïve B cells, switched/non-switched-memory B cells), CD4^+^ T cells (Treg, exhausted PD1 ^+^CD57^−^, naïve CD45RA^+^ CD45RO^−^CD27^+^), CD8^+^ T cells, follicular helper T cells, NK cells (68 subsets total quantified by flow cytometry)	Circulating plasma cells and regulatory B cells (Breg) were selectively and significantly increased in MN patients vs. both CKD and HCs (*p* < 0.025). Plasma cell percentage positively correlated with serum anti-PLA2R1 IgG titers. PLA2R1-specific IgG-producing plasmablasts were detectable in circulation of anti-PLA2R1^+^ MN patients ex vivo. CCR4^+^CD45RA^−^ Treg (high-regulatory-function subset) were significantly lower in MN vs. controls. Naïve CD4^+^ T cells were higher in MN vs. CKD only. No significant differences in follicular helper T cells or intracellular IFN-γ, IL-4, or IL-17 across groups. TNF-α was the only cytokine significantly elevated in MN vs. healthy controls.	68 cell subsets analyzed; after Holm–Bonferroni adjustment, only plasma cells and Breg remained distinctly significant for MN. Random forest and LASSO analyses confirmed plasma cells as the top discriminating subset. Many immunological differences between MN and healthy controls were shared with non-immune CKD patients, suggesting some immune dysregulation is a common CKD feature regardless of etiology. Breg increase interpreted as a counter-regulatory response rather than a primary pathogenic mechanism.
Cheddadi et al., 2026 [13]	adults with PLA2R1-positive pMN treated with RTX	B cells: CD19^+^ total, naïve (CD19^+^CD27^−^IgD^+^), non-switched-memory (CD19^+^CD27^+^IgD^+^), switched-memory (CD19^+^CD27^+^IgD^−^), double-negative (CD19^+^CD27^−^IgD^−^); CD38^+^ subsets: transitional (CD38^++^CD27^−^IgD^+^), plasmablasts (CD38^++^CD27^+^IgD^−^), double-negative CD38^+^ (CD38^++^CD27^−^IgD^−^)	Complete depletion of all B cell subpopulations at M3 post-RTX in all patients. CD19^+^ cells reappeared at M6, predominantly naïve cells, followed by CD38^+^, transitional, and memory cells. At M6, total CD19^+^, naïve, double-negative CD19^+^, and CD38^+^ cells were significantly higher in relapsers vs. nonrelapsers (CD19^+^: 0.80% vs. 0.15%, *p* = 0.005; naïve: 0.47% vs. 0.04%, *p* = 0.008; CD38^+^: 0.35% vs. 0.06%, *p* = 0.008). Memory cell reconstitution occurred earlier in relapsers; switched-memory cells were higher during relapse than remission.	First study to demonstrate the prognostic value of B cell subpopulations in predicting relapse in MN. Optimal cut-offs at M6 for relapse prediction: CD19^+^ ≥ 0.44%, naïve ≥ 0.18%, double-negative CD19^+^ ≥ 0.028%, CD38^+^ ≥ 0.12%, transitional CD38^+^ ≥ 0.072%, double-negative CD38^+^ ≥ 0.024%. Plasmablast count correlated significantly with anti-PLA2R1 titer at M12 (*p* = 0.015).
Gaggar et al., 2023 [14]	20 patients with biopsy-proven pMN (PLA2R1-positive), treatment-naïve and treatment-resistant, treated with RTX	B cells (CD19^+^/CD20^+^); monitored via CD19^+^ B cell count	Low-dose rituximab (500 mg IV × 2 doses, 1 month apart) achieved 66.7% composite remission (CR 5.6% + PR 61.1%) at 12 months. All patients achieved CD19^+^ B cell depletion post-infusion; sustained depletion at 6 months in 84.3% and at 12 months in only 32%. No statistically significant association between B cell depletion and remission was observed (OR 2.25, 95% CI 0.18–27.7; *p* = 0.66). PLA2R1-Ab levels at 12 months were significantly lower in remitters vs. non-remitters (17.8 ± 21.2 vs. 311.7 ± 356.0 RU/mL; *p* = 0.01).	Treatment-naïve patients had higher remission rates than treatment-resistant (85% vs. 54.5%), though not statistically significant. A proportion of 45% adverse events was observed (15% grade ≥ 3, including 2 deaths from sepsis). Authors suggest rituximab dosing should be guided by PLA2R1-Ab kinetics rather than CD19 B cell depletion. Larger BSA was associated with failure to achieve deep B cell depletion (1.74 vs. 1.62 m^2^, *p* = 0.305).
Duan et al., 2026 [15]	149 adult MN patients with anti-PLA2R1 antibody ≥ 2 RU/mL, treated with RTX	B lymphocytes (CD19^+^); neutrophils, monocytes and lymphocytes (as composite indices: NLR, MLR, SIRI)	Short-term (6-month remission): At 3 months, SIRI ≤ 1.25 [OR 3.68 (95% CI 1.39–9.72)] and B cell proportion ≤ 0.2% [OR 2.90 (95% CI 1.00–8.35)] independently predicted 6-month remission; combining these with traditional markers (proteinuria, albumin, anti-PLA2R1) improved AUC from 0.81 to 0.86. Long-term (12-month remission)**:** At 6 months, only SIRI ≤ 0.9 [OR 4.84 (95% CI 1.43–16.40)] and albumin change [OR 1.11 (95% CI 1.03–1.19)] independently predicted 12-month remission.	Non-responders exhibited persistently elevated NLR, MLR, and SIRI throughout the entire 12-month follow-up. SIRI integrates innate and adaptive immune signals; elevated SIRI reflects amplified systemic inflammation with concomitant immunosuppression. B cell depletion serves as an upstream, early predictive event (3 months), while SIRI captures sustained inflammatory activity relevant to long-term prognosis. NLR and MLR did not retain independent predictive value in multivariate analyses. B cells were reported only as a percentage of CD19-positive lymphocytes, with no denominator specified and no absolute counts; no B cell subsets were measured.
Hou et al., 2018 [16]	27 newly diagnosed early-stage pMN patients (stages I–II) and 16 HCs, treated with tacrolimus and prednisone	Monocytes: M1-like (CD14^+^CD163^−^) and M2-like (CD14^+^CD163^+^) monocytes; M2 subsets: CD14^+^CD163^+^CD206^+^, CD14^+^CD163^+^CD115^+^, CD14^+^CD163^+^CD206^+^CD115^+^, and IL-10^+^ M2 monocytes	CD14^+^CD163^+^ M2 monocyte counts were significantly higher in pMN vs. HCs (*p* = 0.021); no significant difference in M1-like monocytes. CD14^+^CD163^+^CD206^+^ M2 counts increased in parallel with proteinuria severity. CD14^+^CD163^+^CD206^+^ cells positively correlated with 24 h urinary protein (R = 0.55), 24 h urinary albumin (R = 0.63), and serum anti-PLA2R1 (R = 0.39). IL-10^+^ M2 monocytes and serum IL-10 were elevated in pMN vs. HCs (*p* < 0.05). After 12 weeks of prednisolone + tacrolimus therapy, M2 subset counts remained higher than HCs despite partial proteinuria remission.	CD14^+^CD163^+^CD206^+^ M2-like monocytes may reflect a Th2-driven immune response (IL-4/IL-13 stimulation) leading to macrophage infiltration of tubulointerstitial lesions, with potential pro-fibrotic effects via fibronectin secretion. Authors propose CD14^+^CD163^+^CD206^+^ M2 monocytes as sensitive biomarkers for pMN disease severity.
Kazan & Kazan 2023 [17]	28 Patients with idiopathic MN (IMN) at low and moderate risk, diagnosed January 2015–January 2022, reviewed retrospectively; treatment: conservative (supportive) therapy only for 6 months—no immunosuppression	No subset immunophenotyping. Composite indices from the routine differential count: systemic immune-inflammation index (SII) and pan-immune-inflammation value (PIV) (neutrophils, lymphocytes, monocytes, platelets)	Patients in the non-remission group had significantly higher SII and PIV than those achieving complete remission (proteinuria < 0.3 g/day and albumin >3.5 g/dL at 6 months) (*p* < 0.05). SII ≥ 1056.2 predicted non-remission with 63.6% sensitivity and 100% specificity; PIV ≥ 447.4 with 100% sensitivity and 70.6% specificity.	Retrospective, single-center; cut-offs derived by ROC analysis within the same cohort, without external validation. Remission was assessed after conservative treatment in low-/moderate-risk disease, so these indices predict spontaneous remission rather than response to immunosuppression, and the cut-offs are not transferable to the RTX-treated setting. Uses SII/PIV rather than SIRI, so the values are not directly comparable with the SIRI cut-offs of Duan et al. [18].
Liang et al., 2026 [18]	187 patients with IMN, prospective observational cohort (February 2022–February 2024), outcome assessed one year after treatment; treatment as per standard practice, not an RTX-specific cohort	No subset immunophenotyping. Composite indices: SII, SIRI, PIV and lymphocyte-to-monocyte ratio (LMR)	Non-remission patients showed significantly higher SII, SIRI and PIV, and lower eGFR and LMR. On multivariate logistic regression, hypertension, 24 h urinary protein, SII, SIRI, and PIV were independent risk factors for non-remission, while eGFR was protective. ROC: SII AUC 0.743; PIV AUC 0.759.	The largest prospective cohort supporting composite innate-inflammatory indices as prognostic markers in IMN, and independent of the RTX-treated Duan cohort [18]—providing external, though not RTX-specific, support for the prognostic relevance of SIRI. Indices derive from routine hematology and therefore reflect systemic inflammation rather than a defined cell subset. Because treatment was not RTX-based, these findings speak to prognosis in IMN generally rather than to pharmacodynamic response to B cell depletion.
Pozdzik et al., 2016 [19]	Single adult patient (*n* = 1) with anti-PLA2R1 antibody-related MN, treated with RTX, followed for 4 years (single case report)	Flow cytometry: plasmablasts (CD3^−^CD19^+^CD20^−^IgD^−^CD27^high^CD38^high^); memory B cells (CD3^−^CD19^+^CD20^+^IgD^−^CD27^+^CD38^−^); naïve B cells (CD3^−^CD19^+^CD20^+^IgD^+^CD27^−^CD38^low^)	RTX produced complete disappearance of CD19^+^ B cells, plasmablasts and memory B cells by day 15. Despite persisting CD19^+^ lymphopenia, plasmablasts and memory B cells reappeared before naïve B cells (days 45, 90, and 120, respectively). During follow-up, plasmablasts declined faster than memory B cells but remained above the pre-RTX (day 0) level, while anti-PLA2R1 antibody rose progressively.	Single-patient observation; hypothesis-generating only, no statistical inference possible. Provides the only direct longitudinal description in anti-PLA2R1-related MN of CD20-negative plasmablast re-emergence during ongoing CD19^+^ depletion, and of the temporal association between persistent plasmablasts and rising anti-PLA2R1 antibody. Cited here as a mechanistic illustration of CD20-independent autoantibody production, not as evidence of predictive value.
Ramachandran et al., 2020 [20]	24 adult patients with biopsy-proven primary membranous nephropathy (pMN) treated with cyclophosphamide/glucocorticoids (CYC/GC); 10 healthy controls (HCs—voluntary kidney donors, matched for age and sex)	T regulatory cells (TREG: CD3^+^CD4^+^CD25^hi^CD127^low^FoxP3^+^); B regulatory cells (BREG: CD19^+^CD5^+^CD1d^hi^IL-10^+^)	Baseline: BREG significantly lower in pMN patients vs. HCs (*p* = 0.0007); TREG trend toward lower levels but not significant (*p* = 0.07). Post-treatment (month 8): Significant increase in both BREG (*p* = 0.001) and TREG (*p* = 0.02) compared with baseline. Responders vs. non-responders: BREG significantly increased at months 6 and 8 in responders (*p* < 0.001) but not in non-responders; TREG increased in responders at month 8 (*p* = 0.05).	PLA2R1-related pMN in 79% of patients. No association between anti-PLA2R antibody titers and TREG or BREG levels at baseline. CYC, known to suppress TREG, addressed by a 2-month washout period before the month 8 sample. On multivariate analysis, BREG showed a trend toward association with outcome (*p* = 0.09) but did not reach significance, likely limited by small sample size (*n* = 24). First study to evaluate BREG in pMN.
Rosenzwajg et al., 2017 [11]	Humans—25 patients with pMN and 27 healthy donors (HDs), treated with RTX	B cells (CD19^+^), T cells (CD4^+^, CD8^+^), Treg cells (CD4^+^CD25^hi^CD127^−^/^lo^FoxP3^+^), NK cells (CD3^−^CD56^+^), CD56^bright^CD16^−^/^lo^ NK cells	Baseline (pMN vs. HD): ↓ switched (IgD^−^CD27^+^) and nonswitched (IgD^+^CD27^+^) memory B cells; ↑ naïve (IgD^+^CD27^−^) and double-negative (IgD^−^CD27^−^) B cells; ↓ Treg cell frequency among CD4^+^ T cells (3.2% vs. 4.7%, *p* < 0.0001); ↑ effector memory CD4^+^ and CD8^+^ T cells; ↓ total NK cells with ↑ CD56^bright^CD16^−^/^lo^ NK subset. Post-rituximab (responders only): Progressive ↑ in Treg cell % as early as day 8, sustained to month 6; ↑ total NK cells and ↓ CD56^bright^ NK cells; B cell (CD19^+^) depletion correlated with Treg cell increase (Spearman *r*, *p* = 0.04). Cytokines: ↑ IL-5, TNF-α, IL-2RA and ↓ IL-17, IL-1α, IL-7, GM-CSF at baseline vs. HD; TNF-α decreased after rituximab.	Ancillary study to the GEMRITUX RCT (NCT01508468). Sixteen patients received supportive therapy + rituximab and 9 received supportive therapy only. Treg cell % at baseline was significantly lower in rituximab responders vs. non-responders (2.4% vs. 3.9%, *p* = 0.01), suggesting baseline low Treg levels may predict rituximab response. Absolute Treg cell numbers were comparable at baseline between groups. Follow-up limited to 6 months.
Yu et al., 2025 [21]	Bidirectional two-sample Mendelian randomization using published GWAS summary statistics for 731 immune cell traits and for MN; no patients treated—genetic association study	731 immunophenotypes (B cell, T cell, Treg, monocyte, myeloid and dendritic-cell traits)	Inverse-variance weighting identified positive associations with MN for 17 immune cell subtypes (*p* < 0.05, OR > 1) and negative associations for 29 subtypes (*p* < 0.05, OR < 1). Reverse MR identified 2 positively and 4 negatively associated subtypes. No evidence of horizontal pleiotropy (*p* > 0.05); MR-Egger, weighted-median and weighted-mode analyses were concordant.	Offers genetic, treatment-independent support for a causal contribution of specific immune cell traits to MN susceptibility, complementing the observational immunophenotyping data. Significance was declared at a nominal *p* < 0.05 across 731 traits tested, so individual subtype associations should be treated as exploratory pending replication. Provides no information on response to RTX.
Zhang et al., 2024 [22]	58 pMN patients and 25 HCs; 40 patients received RTX and were followed up for ≥6 months (avg. 14.5 months)	CD3^+^ T cells, CD4^+^ T cells, CD8^+^ T cells, CD4^+^ central memory T cells (CD4^+^CCR7^+^CD45RA^−^), CD4^+^ effector memory T cells (CD4^+^CCR7^−^CD45RA^−^), CD8^+^ central memory T cells, CD8^+^ effector memory T cells, Treg cells (CD4^+^CD25^+^CD127^lo^), CD4^+^CD25^+^ T cells (IL-2Rα), DNT cells (CD3^+^CD4^−^CD8^−^), NK cells (CD16^+^CD56^+^), B cells (CD3^−^CD19^+^)	At baseline vs. healthy controls: CD3^+^, CD4^+^, CD4^+^ central memory, CD4^+^ effector memory T cells, CD4^+^CD25^+^ T cells (IL-2Rα), and CD4^+^/CD8^+^ ratio were significantly elevated in pMN patients. CD8^+^ T cells (total, central memory, effector memory) and Treg cells (CD4^+^CD25^+^CD127^lo^) were significantly reduced. No difference in DNT cells or NK cells. After RTX treatment (6 months): Treg cells significantly increased (*p* = 0.019); CD4^+^CD25^+^ T cells and CD4^+^/CD8^+^ ratio significantly decreased (*p* = 0.016 and *p* = 0.002, respectively). Total T cell, CD4^+^, CD8^+^, memory T cell subsets and NK cell counts were not significantly affected by RTX.	RTX-induced changes in Treg cells and CD4^+^CD25^+^ T lymphocytes were not correlated with B cell counts or anti-PLA2R1 antibody titers, suggesting an independent mechanism of T cell subset restoration by RTX, separate from B cell depletion. Response rate at 6 months: 72.5% (partial + complete remission). B cell depletion confirmed by significant reduction in CD3^−^CD19^+^ cells post-RTX (*p* < 0.001).
Deng et al., 2023 [23]	pMN patients vs. HCs; subgroup of pMN patients receiving cyclophosphamide/corticosteroids	CD19^+^CD24^hi^CD38^hi^ B cells (regulatory B cells/Bregs); Th2 cells; Th17 cells	Increased frequency of CD19^+^CD24^hi^CD38^hi^ B cells in pMN vs. HCs. Frequency positively correlated with 24h urinary protein, negatively correlated with serum total protein and albumin. CD19^+^CD24^hi^CD38^hi^ B cells displayed a pro-inflammatory skew (↑ IL-6, ↑ IL-12, ↓ IL-10). Th2 and Th17 cells were upregulated; CD19^+^CD24^hi^CD38^hi^ frequency positively correlated with Th17 frequency. After 4 weeks of cyclophosphamide, CD19^+^CD24^hi^CD38^hi^ B cell percentage significantly decreased.	CD19^+^CD24^hi^CD38^hi^ B cells in pMN are functionally impaired—despite expansion, they lose their canonical immunosuppressive IL-10 production and instead adopt a pro-inflammatory cytokine profile (↑ IL-6, ↑ IL-12). This functional switch may contribute to IMN pathogenesis. Positive association with Th17 cells suggests a Breg–Th17 axis in disease activity.

Abbreviations: NLR: neutrophil-to-lymphocyte ratio, MLR: monocyte-to-lymphocyte ratio, SIRI: systemic inflammation response index, pMN: primary membranous nephropathy, HCs: healthy controls.

**Table 2:** Classification of the peripheral immune parameters discussed in this review, by marker category, evidence source and readiness tier in rituximab-treated pMN.

Subset/Parameter	Marker Class	Tier	Evidence Source	Basis for the Assignment
Total CD19+ B cells (depletion at month 3)	Pharmacodynamic	A	Direct (RTX-treated pMN)	Direct pharmacodynamic readout; failure to deplete has an actionable interpretation (underdosing, urinary loss, anti-rituximab antibodies)
B cell reconstitution profile at month 6 (total CD19+, naïve, transitional, CD38+, double-negative, class-switched memory)	Relapse	B	Direct (one RTX-treated pMN cohort); supportive data extrapolated from INS	Higher month-6 frequencies of total CD19+, naïve, double-negative and CD38+ cells associated with subsequent relapse in a single cohort; ROC-derived cut-offs, no external validation
Class-switched-memory B cells (CD19+CD27+IgD−), sustained depletion during remission	Disease activity	B	Extrapolated (pediatric INS, other anti-CD20-treated autoimmune disease); limited direct pMN	Consistent depletion in responders; premature return associated with relapse in related diseases, but not shown to outperform other subsets in pMN
Plasmablasts *	Disease activity; mechanism of anti-CD20 failure	B	Direct (single-patient kinetics and small pMN cohorts)	Mechanistically decisive for anti-CD20 failure; longitudinal data confined to single-patient and small-cohort studies
Regulatory T cells (CD4+CD25+FoxP3+)	Predictive	B	Direct (one mechanistic pMN cohort)	Early post-treatment rise associated with remission in one mechanistic cohort only; not replicated
CD45RA/CD45RO axis in CD4+ T cells	Disease activity	B	Direct (cross-sectional pMN)	Reproducible skewing toward memory in active disease; no demonstrated predictive value
SIRI and related composite indices	Prognostic—derived from the routine differential count, not immunophenotyping	B	Direct (one RTX-treated pMN cohort); indirect (non-RTX MN cohorts)	Associated with remission in one 149-patient cohort; cut-offs are cohort-specific ROC-derived and unreplicated
Non-switched-memory B cells (CD27+IgM+IgD+)	Disease activity	C	Direct (cross-sectional pMN)	Reduced at baseline; no outcome association reported
Regulatory B cells (CD5+CD1d^hi^, CD24-hi CD38^hi^)	Disease activity	C	Direct (small cross-sectional pMN cohorts)	Conflicting phenotype definitions; no longitudinal data under anti-CD20 in pMN
CD4/CD8 ratio	Descriptive only	C	Direct (conflicting pMN cohorts)	Direction of change disputed between cohorts; not interpretable in isolation
Monocyte subsets (classical/intermediate/non-classical)	Descriptive only	C	Extrapolated (CKD, IgA nephropathy)	Mostly extrapolated from CKD and IgA nephropathy; single small pMN cohort
M2-like CD14+CD163+CD206+ monocytes	Disease activity	C	Indirect (pMN, non-RTX-treated)	Correlate with proteinuria at baseline but did not change with effective therapy

Marker categories and tiers are defined in Section 1.2. Evidence source: direct = RTX-treated pMN cohorts; indirect = pMN cohorts that were not RTX-treated or were cross-sectional; extrapolated = data from other immune-mediated diseases. No parameter listed has been prospectively validated for clinical use. * Bone marrow long-lived plasma cells are mechanistically decisive for anti-CD20 failure, but inaccessible without bone marrow sampling, therefore not a candidate biomarker and not tiered.

**Table 3:** B-Lymphocyte compartment in pMN treated with RTX.

B Cell Subset	Phenotype	Role in pMN	Effect of RTX	Clinical Significance
Total B cells	CD3−CD19+	Humoral immune orchestrator; potent antigen-presenting cell; provides necessary B cell help for maintenance of autoimmune response.	Rapid and profound depletion to <5 cells/µL within 2 weeks; CD19+ depletion often sustained for 6–9 months in majority of patients.	Complete CD19+ depletion confirms target engagement and pharmacodynamic efficacy; however, CD19+ depletion alone does not reliably predict clinical response and should be complemented by serological (anti-PLA2R1) monitoring.
Naïve B cells	CD19+CD27−IgD+(IgM+)	Antigen-inexperienced bone marrow emigrants; possess broad repertoire and low affinity for self-antigens.	Depleted by day 15; repopulation begins ~45–120 days post-infusion; dominates the repopulation phase at months 6–12 with gradually rising absolute counts.	Progressive naïve B cell reconstitution is considered favorable and, together with delayed memory B cell recovery, has been associated with sustained remission in RTX-treated nephrotic syndromes.
Class-switched memory B Cells	CD19+CD27+IgD−(primarily IgG4+)	Direct precursor to pathogenic IgG4 anti-PLA2R1-secreting plasma cells; retain high-affinity and class-switched specificities; perpetuate autoimmune responses.	Depleted rapidly by day 15; depletion is more complete and repopulation more delayed in responders than in non-responders; earlier and more pronounced reconstitution (e.g., within the first year) has been associated with higher relapse risk in RTX-treated nephrotic syndromes.	Early re-emergence of CD27+IgD− memory B cells has been associated with relapse in small series and in related anti-CD20-treated diseases. The IgG4-committed fraction is a minority of this compartment and is not resolved by standard clinical panels.
Plasmablasts and long-lived plasma cells	CD19+CD20−IgD−CD27^hi^CD38^hi^ (circulating plasmablasts); CD138+CD38+CD19−/low (bone marrow LLPCs)	Terminal antibody-secreting effectors; immediate, high-output source of pathogenic IgG4 anti-PLA2R1 autoantibodies; LLPCs maintain long-lived antibody secretion within bone marrow niches that is relatively independent of short-term peripheral CD20+ B cell dynamics.	Not directly depleted by RTX due to complete absence of CD20 surface expression; circulating plasmablasts transiently decrease as upstream precursor (memory B cell) supply is interrupted, but re-emerge as early as days 45–90 post-infusion in some patients; LLPCs largely unaffected by CD20+ depletion.	Persistence of elevated plasmablasts despite complete CD19+ depletion is a plausible and increasingly recognized mechanism contributing to RTX non-response in some patients. Not accessible to anti-CD20 therapy of either type, as neither plasmablasts nor long-lived plasma cells express CD20. Type II anti-CD20 antibodies such as obinutuzumab act upstream, through deeper and more sustained depletion of the CD20+ precursor pool that replenishes the antibody-secreting compartment, whereas direct targeting requires plasma-cell-directed agents such as the anti-CD38 antibody felzartamab.
Non-switched memory B cells	CD19+CD27+IgD+(IgM+)	Marginal zone-like B cells; mediate rapid, early T cell-independent activation responses and early innate-like recall.	Depleted rapidly; repopulation kinetics similar to class-switched memory; modest delay when compared with naïve B cells.	Persistent or early re-expansion of memory subsets (both switched and non-switched) suggests residual immunologic memory; however, the most reliable prognostic data involve the switched-memory compartment.
Regulatory B cells	CD19+CD5+CD1d^hi^	Tolerance enforcers via IL-10 and IL-35 secretion; actively suppress effector T cell proliferation and Tfh differentiation; dampen B cell autoimmunity.	RTX transiently depletes Bregs along with other CD19+ subsets; in other glomerular diseases and in pMN treated with alkylating-agent regimens, recovery of functional IL-10-producing Bregs has been associated with disease remission. Whether a similar Breg rebound pattern after RTX specifically predicts outcome in pMN remains to be determined.	A proportional increase in IL-10-competent Bregs, together with Treg recovery, is proposed as a favorable immunologic signature, but its predictive value for long-term remission in RTX-treated pMN is still investigational.

Abbreviations: RTX: rituximab, pMN: primary membranous nephropathy, PLA2R1: phospholipase A2 receptor.

**Table 4:** T-lymphocyte compartment in pMN treated with RTX.

Cell Subset	Phenotype	Role in pMN	Effect of RTX	Clinical Significance
Total T helper cells	CD3+CD4+	Orchestrates autoimmune response; Tfh2 subset drives IgG4 class switching via IL-21/IL-4 and CD40-CD40L.	Reported changes include a fall in the CD4+/CD8+ ratio and in activated CD4+ subsets with a rise in Tregs; these derive from small cohorts and have not been replicated.	Elevated CD4+/CD8+ ratio has been reported in active disease, but pMN cohorts conflict.
Cytotoxic T cells	CD3+CD8+	Contribute to systemic inflammation; exhausted subsets accumulate during chronic autoimmune exposure; there are few pMN-specific data on CD8 exhaustion.	Subset redistribution: CD4+/CD8+ ratio normalization partly reflects relative CD8+ preservation.	Expression of exhaustion markers (e.g., PD-1) may reflect chronic antigen exposure and immune dysregulation; their prognostic value in pMN remains exploratory.
Naïve T helper cells	CD4+CD45RA+	Antigen-inexperienced precursors, which require strong TCR engagement and costimulation for activation.	Proportional recovery suggested in responders; requires longitudinal validation.	Rising proportion has been interpreted as a return toward immunological homeostasis but requires longitudinal validation.
Memory T helper cells	CD4+CD45RO+	Chronically activated effector memory clones with low activation threshold; sustain autoantibody production.	Reduction in percentage and/or absolute numbers has been reported in RTX responders.	Persistent elevation is a candidate biomarker for treatment resistance and relapse risk.
Regulatory T cells	CD4+CD25+FoxP3+ (operationally: CD4+CD25highCD127low/neg)	Enforce peripheral tolerance, suppress effector T cells, and indirectly suppress autoantibody production.	Rapid induction detectable by day 8 in responders in a single cohort.	Low baseline + rapid post-infusion surge is the most frequently cited candidate predictor of early RTX response, based on a single small cohort.

Abbreviations: RTX: rituximab, pMN: primary membranous nephropathy.

**Table 5:** Monocytes in pMN treated with RTX.

Cell Subset	Phenotype	Role in pMN	Effect of RTX	Clinical Significance
Classical	CD14++CD16−	Primary phagocytes; initial responders to acute inflammation via CCR2-dependent recruitment to sites of acute tissue injury.	May change non-specifically with the acute-phase response and resolution of inflammation; no specific RTX-related kinetic pattern has been validated in pMN.	A relative reduction in classical monocytes with expansion of CD16+ subsets has been associated with higher cardiovascular risk in CKD and coronary artery disease; classical monocyte counts alone are not a validated prognostic marker in pMN.
Intermediate	CD14++CD16+	Highly pro-inflammatory subset with strong HLA-DR expression and potent antigen-presenting capacity; produces large amounts of TNF-α, IL-1β, IL-6.	Expanded in proteinuric renal diseases and CKD; global indices reflecting monocyte-driven inflammation (e.g., SIRI) fall in responders and remain elevated in non-responders.	Correlates with systemic inflammatory burden and endothelial dysfunction in IgA nephropathy and progressive CKD; acts as a proxy for systemic inflammation in RTX-treated membranous nephropathy.
Non-classical	CD14+CD16++	“Patrolling” monocytes that survey the vascular endothelium and participate in the resolution of acute inflammation; they overlap phenotypically with the M2-like (CD163/CD206+) subset.	Kinetics after immunosuppressive therapy in pMN have not been systematically characterized; persistent elevation would be expected to reflect ongoing endothelial activation and tissue remodeling.	Conceptually reflects ongoing, low-grade vascular surveillance and tissue repair; sustained expansion of CD16+ (including non-classical) monocytes in CKD is associated with endothelial dysfunction and atherosclerosis, but specific prognostic data in RTX-treated pMN are lacking.
M2-like	CD14+CD163+CD206+	Alternatively activated monocytes with high expression of scavenger receptors CD163 and CD206; produce IL-10 and TGF-β.	Not studied; in early idiopathic MN cohorts treated with calcineurin inhibitors, CD14+CD163+CD206+ monocytes remain elevated despite partial improvement in proteinuria, indicating persistent matrix repair and low-grade fibrotic signaling.	In early idiopathic MN, these cells are expanded and correlate positively with 24 h proteinuria and serum anti-PLA2R1 titers; their expansion at baseline marks more severe immunologic and clinical activity; whether persistence despite clinical response truly signals ongoing glomerulosclerosis and interstitial fibrosis remains to be proven.
Activated monocytes	CD14++HLA-DR+	Denote monocytes with high antigen-presenting activity; high HLA-DR reflects active adaptive immune engagement; very low HLA-DR marks immune paralysis (sepsis, malignancy).	The prognostic value of longitudinal CD14++HLA-DR+ kinetics under RTX in pMN has not been systematically validated; higher monocyte HLA-DR is associated with increased diabetic nephropathy risk.	Mendelian randomization studies suggest that genetically higher monocyte HLA-DR expression increases risk of progressive kidney diseases such as diabetic nephropathy, but similar genetic associations have not yet been shown for pMN. In pMN, monocyte HLA-DR currently reflects general innate activation rather than a validated disease-specific biomarker.
Systemic inflammation response index (SIRI)	(Neutrophils × monocytes)/lymphocytes	Captures the balance between innate effector cells (neutrophils, monocytes) and adaptive lymphocytes; serves as a composite marker of systemic immune inflammation.	In RTX-treated pMN, SIRI scores fall significantly in responders and remain persistently elevated in non-responders; in one prospective cohort, SIRI ≤ 1.25 at month 3 (combined with B cell depletion ≤ 0.2%) predicted 6-month remission, and SIRI ≤ 0.9 at month 6 (with rising albumin) predicted 12-month remission.	In the Duan et al. cohort, adding SIRI and B cell depletion to traditional predictors improved the AUC for 6-month remission from 0.81 to 0.86; by month 6, only SIRI and albumin remained independent predictors of 12-month remission, as anti-PLA2R1 and B cell levels converged between responders and non-responders. Cut-offs are cohort-specific and require external validation.

Abbreviations: RTX: rituximab, pMN: primary membranous nephropathy.

## Data Availability

No new data were created or analyzed in this study. Data sharing is not applicable to this article.

## References

[1] Ronco P., Beck L., Debiec H., Fervenza F.C., Hou F.F., Jha V., Sethi S., Tong A., Vivarelli M., Wetzels J. (2021). Membranous nephropathy. Nat. Rev. Dis. Primers.

[2] Tomas N.M., Beck L.H., Meyer-Schwesinger C., Seitz-Polski B., Ma H., Zahner G., Dolla G., Hoxha E., Helmchen U., Dabert-Gay A.-S. (2014). Thrombospondin Type-1 Domain-Containing 7A in Idiopathic Membranous Nephropathy. N. Engl. J. Med..

[3] Beck L.H., Bonegio R.G.B., Lambeau G., Beck D.M., Powell D.W., Cummins T.D., Klein J.B., Salant D.J. (2009). M-Type Phospholipase A2 Receptor as Target Antigen in Idiopathic Membranous Nephropathy. N. Engl. J. Med..

[4] Seifert L., Zahner G., Meyer-Schwesinger C., Hickstein N., Dehde S., Wulf S., Köllner S.M.S., Lucas R., Kylies D., Froembling S. (2023). The classical pathway triggers pathogenic complement activation in membranous nephropathy. Nat. Commun..

[5] Van der Woude F.J., Rasmussen N., Lobatto S., Wiik A., Permin H., van Es L.A., van der Giessen M., van der Hem G.K., The T.H. (1985). Autoantibodies against neutrophils and monocytes: Tool for diagnosis and marker of disease activity in Wegener’s granulomatosis. Lancet.

[6] (2021). Kidney Disease: Improving Global Outcomes (KDIGO) Glomerular Diseases Work Group. KDIGO 2021 Clinical Practice Guideline for the Management of Glomerular Diseases. Kidney Int..

[7] Dumoulin A., Hill G.S., Montseny J.J., Meyrier A. (2003). Clinical and morphological prognostic factors in membranous nephropathy: Significance of focal segmental glomerulosclerosis. Am. J. Kidney Dis..

[8] Fervenza F.C., Appel G.B., Barbour S.J., Rovin B.H., Lafayette R.A., Aslam N., Jefferson J.A., Gipson P.E., Rizk D.V., Sedor J.R. (2019). Rituximab or Cyclosporine in the Treatment of Membranous Nephropathy. N. Engl. J. Med..

[9] Gauckler P., Shin J.I., Alberici F., Audard V., Bruchfeld A., Busch M., Cheung C.K., Crnogorac M., Delbarba E., Eller K. (2021). Rituximab in Membranous Nephropathy. Kidney Int. Rep..

[10] Fervenza F.C., Hou F.F., Hao C.-M., Kirsztajn G.M., Gesualdo L., Hryszko T., Pisani A., Roccatello D., Bomback A.S., Rae J. (2026). Obinutuzumab or Tacrolimus in Primary Membranous Nephropathy. N. Engl. J. Med..

[11] Rosenzwajg M., Languille E., Debiec H., Hygino J., Dahan K., Simon T., Klatzmann D., Ronco P. (2017). B- and T-cell subpopulations in patients with severe idiopathic membranous nephropathy may predict an early response to rituximab. Kidney Int..

[12] Cantarelli C., Jarque M., Angeletti A., Manrique J., Hartzell S., O’Donnell T., Merritt E., Laserson U., Perin L., Donadei C. (2020). A Comprehensive Phenotypic and Functional Immune Analysis Unravels Circulating Anti–Phospholipase A2 Receptor Antibody Secreting Cells in Membranous Nephropathy Patients. Kidney Int. Rep..

[13] Cheddadi Y., El Maï M., Brglez V., Nahon Carzo S., Cremoni M., Teisseyre M., Seitz-Polski B. (2026). Early-Stage B-cells Predict Relapse After Rituximab Treatment in Patients With Membranous Nephropathy. Kidney Int. Rep..

[14] Gaggar P., Madipally R., Raju S.B. (2023). Rituximab, Use and B Cell Depletion in Patients with Membranous Nephropathy- A Retrospective, Observational Study. Indian J. Nephrol..

[15] Duan S., Ye Y., Zhou Q., Hua H., Zeng M., Zhang C., Yuan Y., Xing C., Mao H., Zhang B. (2026). Systemic inflammation and B cell indices predict rituximab responses in membranous nephropathy. Clin. Kidney J..

[16] Hou J., Zhang M., Ding Y., Wang X., Li T., Gao P., Jiang Y. (2018). Circulating CD14+ CD163+ CD206+ M2 Monocytes Are Increased in Patients with Early Stage of Idiopathic Membranous Nephropathy. Mediat. Inflamm..

[17] Kazan D.E., Kazan S. (2023). Systemic immune inflammation index and pan-immune inflammation value as prognostic markers in patients with idiopathic low and moderate risk membranous nephropathy. Eur. Rev. Med. Pharmacol. Sci..

[18] Liang F., Yang Y., Sun Y., Xing L., Yu X., Xia J., Gao J. (2026). Association of Systemic Immune Inflammation Index and Pan-immune Inflammation Value with Prognosis in Idiopathic Membranous Nephropathy. Iran. J. Allergy Asthma Immunol..

[19] Pozdzik A., Beukinga I., Gu-Trantien C., Willard-Gallo K., Nortier J., Pradier O. (2016). Circulating (CD3− CD19+ CD20− IgD− CD27high CD38high) Plasmablasts: A Promising Cellular Biomarker for Immune Activity for Anti-PLA2R1 Related Membranous Nephropathy?. Mediat. Inflamm..

[20] Ramachandran R., Kaundal U., Girimaji N., Rakha A., Rathi M., Gupta K.L., Kohli H.S., Jha V. (2020). Regulatory B Cells Are Reduced and Correlate with Disease Activity in Primary Membranous Nephropathy. Kidney Int. Rep..

[21] Yu Y., Liu G., Zhang R., Chen B., Luan Z. (2025). Association between immune cell subtypes and membranous nephropathy: A bidirectional Mendelian randomization study. Medicine.

[22] Zhang Y., Yang J., Li J., Sun J., Zhou L., Xu D., Sha W., Dai L., Shen L. (2024). Rituximab may affect T lymphocyte subsets balance in primary membranous nephropathy. BMC Nephrol..

[23] Deng B., Deng L., Liu M., Zhao Z., Huang H., Tu X., Liang E., Tian R., Wang X., Wang R. (2023). Elevated circulating CD19+CD24hiCD38hi B cells display pro-inflammatory phenotype in idiopathic membranous nephropathy. Immunol. Lett..

[24] Rastogi I., Jeon D., Moseman J.E., Muralidhar A., Potluri H.K., McNeel D.G. (2022). Role of B cells as antigen presenting cells. Front. Immunol..

[25] Velounias R.L., Tull T.J. (2022). Human B-cell subset identification and changes in inflammatory diseases. Clin. Exp. Immunol..

[26] Basu B., Angeletti A., Islam B., Ghiggeri G.M. (2022). New and Old Anti-CD20 Monoclonal Antibodies for Nephrotic Syndrome. Where We Are?. Front. Immunol..

[27] Del Vecchio L., Allinovi M., Rocco P., Brando B. (2021). Rituximab Therapy for Adults with Nephrotic Syndromes: Standard Schedules or B Cell-Targeted Therapy?. J. Clin. Med..

[28] Boyer-Suavet S., Andreani M., Lateb M., Savenkoff B., Brglez V., Benzaken S., Bernard G., Nachman P.H., Esnault V., Seitz-Polski B. (2020). Neutralizing Anti-Rituximab Antibodies and Relapse in Membranous Nephropathy Treated With Rituximab. Front. Immunol..

[29] Morbach H., Eichhorn E.M., Liese J.G., Girschick H.J. (2010). Reference values for B cell subpopulations from infancy to adulthood. Clin. Exp. Immunol..

[30] So B.Y.F., Yap D.Y.H., Chan T.M. (2021). B Cells in Primary Membranous Nephropathy: Escape from Immune Tolerance and Implications for Patient Management. Int. J. Mol. Sci..

[31] Colucci M., Carsetti R., Cascioli S., Casiraghi F., Perna A., Ravà L., Ruggiero B., Emma F., Vivarelli M. (2016). B Cell Reconstitution after Rituximab Treatment in Idiopathic Nephrotic Syndrome. J. Am. Soc. Nephrol..

[32] Chen Z., Xu Q., Shou Z. (2024). Application of CD38 monoclonal antibody in kidney disease. Front. Immunol..

[33] Weller S., Braun M.C., Tan B.K., Rosenwald A., Cordier C., Conley M.E., Plebani A., Kumararatne D.S., Bonnet D., Tournilhac O. (2004). Human blood IgM “memory” B cells are circulating splenic marginal zone B cells harboring a prediversified immunoglobulin repertoire. Blood.

[34] She Z., Li C., Wu F., Mao J., Xie M., Hun M., Abdirahman A.S., Luo S., Wan W., Tian J. (2022). The Role of B1 Cells in Systemic Lupus Erythematosus. Front. Immunol..

[35] Long W., Zhang H., Yuan W., Lan G., Lin Z., Peng L., Dai H. (2021). The Role of Regulatory B cells in Kidney Diseases. Front. Immunol..

[36] Yang B., Tan X., Dong S., Wang M., Wu D., Zhang G., Chen X., Wang M., Yang H., Li Q. (2025). The Role of IL-10-Producing Regulatory B Cells in Children With Primary Nephrotic Syndrome. Nephrology.

[37] Chung E.Y.M., Wang Y.M., Keung K., Hu M., McCarthy H., Wong G., Kairaitis L., Bose B., Harris D.C.H., Alexander S.I. (2022). Membranous nephropathy: Clearer pathology and mechanisms identify potential strategies for treatment. Front. Immunol..

[38] Zhao Q., Dai H., Liu X., Jiang H., Liu W., Feng Z., Zhang N., Gao Y., Dong Z., Zhou X. (2021). Helper T Cells in Idiopathic Membranous Nephropathy. Front. Immunol..

[39] Cremoni M., Brglez V., Perez S., Decoupigny F., Zorzi K., Andreani M., Gérard A., Boyer-Suavet S., Ruetsch C., Benzaken S. (2020). Th17-Immune Response in Patients With Membranous Nephropathy Is Associated With Thrombosis and Relapses. Front. Immunol..

[40] Al Barashdi M.A., Ali A., McMullin M.F., Mills K. (2021). Protein tyrosine phosphatase receptor type C (PTPRC or CD45). J. Clin. Pathol..

[41] Courtney A.H., Shvets A.A., Lu W., Griffante G., Mollenauer M., Horkova V., Lo W.-L., Yu S., Stepanek O., Chakraborty A.K. (2019). CD45 functions as a signaling gatekeeper in T cells. Sci. Signal..

[42] Schmidt A., Oberle N., Krammer P.H. (2012). Molecular Mechanisms of Treg-Mediated T Cell Suppression. Front. Immunol..

[43] Xie W.W., Huang J.B., Zhou Y.C., Yuan J.Y., Feng J.X., Shi X.H., Tian L., Zeng X.H., Qiu S.Q., Zhao M.Z. (2026). The immunobiology and therapeutic potential of regulatory T cells in autoimmune diseases and allergic diseases. Front. Immunol..

[44] Kapellos T.S., Bonaguro L., Gemünd I., Reusch N., Saglam A., Hinkley E.R., Schultze J.L. (2019). Human Monocyte Subsets and Phenotypes in Major Chronic Inflammatory Diseases. Front. Immunol..

[45] Ziegler-Heitbrock L., Hofer T.P.J. (2013). Toward a refined definition of monocyte subsets. Front. Immunol..

[46] Dounousi E., Duni A., Naka K.K., Vartholomatos G., Zoccali C. (2021). The Innate Immune System and Cardiovascular Disease in ESKD: Monocytes and Natural Killer Cells. Curr. Vasc. Pharmacol..

[47] Duni A., Kitsos A., Bechlioulis A., Markopoulos G.S., Lakkas L., Baxevanos G., Mitsis M., Vartholomatos G., Naka K.K., Dounousi E. (2023). Differences in the Profile of Circulating Immune Cell Subsets in Males with Type 2 Cardiorenal Syndrome versus CKD Patients without Established Cardiovascular Disease. Biomedicines.

[48] Duni A., Vartholomatos G., Balafa O., Ikonomou M., Tseke P., Lakkas L., Rapsomanikis K.P., Kitsos A., Theodorou I., Pappas C. (2021). The Association of Circulating CD14++CD16+ Monocytes, Natural Killer Cells and Regulatory T Cells Subpopulations With Phenotypes of Cardiovascular Disease in a Cohort of Peritoneal Dialysis Patients. Front. Med..

[49] Heine G.H., Ortiz A., Massy Z.A., Lindholm B., Wiecek A., Martínez-Castelao A., Covic A., Goldsmith D., Süleymanlar G., London G.M. (2012). Monocyte subpopulations and cardiovascular risk in chronic kidney disease. Nat. Rev. Nephrol..

[50] Dregoesc M.I., Ţigu A.B., Bekkering S., van der Heijden C.D.C.C., Rodwell L., Bolboacă S.D., Joosten L.A.B., Netea M.G., Riksen N.P., Iancu A.C. (2024). Intermediate monocytes are associated with the first major adverse cardiovascular event in patients with stable coronary artery disease. Int. J. Cardiol..

[51] Lee J., Tam H., Adler L., Ilstad-Minnihan A., Macaubas C., Mellins E.D. (2017). The MHC class II antigen presentation pathway in human monocytes differs by subset and is regulated by cytokines. PLoS ONE.

[52] Thaler B., Hohensinner P.J., Krychtiuk K.A., Matzneller P., Koller L., Brekalo M., Maurer G., Huber K., Zeitlinger M., Jilma B. (2016). Differential in vivo activation of monocyte subsets during low-grade inflammation through experimental endotoxemia in humans. Sci. Rep..

[53] Cormican S., Negi N., Naicker S.D., Islam M.N., Fazekas B., Power R., Griffin T.P., Dennedy M.C., MacNeill B., Malone A.F. (2023). Chronic Kidney Disease Is Characterized by Expansion of a Distinct Proinflammatory Intermediate Monocyte Subtype and by Increased Monocyte Adhesion to Endothelial Cells. J. Am. Soc. Nephrol..

[54] Sendic S., Mansouri L., Lundberg S., Nopp A., Jacobson S.H., Lundahl J. (2021). B cell and monocyte phenotyping: A quick asset to investigate the immune status in patients with IgA nephropathy. PLoS ONE.

[55] Tie X., Chen Z., Yao S., Wu B., Yan B., Zhai H., Qiao X., Su X., Wang L. (2025). Immune Imbalance in Primary Membranous Nephropathy at Single-cell Resolution. Front. Biosci. Landmark.

[56] Buscher K., Marcovecchio P., Hedrick C.C., Ley K. (2017). Patrolling Mechanics of Non-Classical Monocytes in Vascular Inflammation. Front. Cardiovasc. Med..

[57] Wang Y., Fu S., Su S., Xu Z. (2025). Monocyte human leukocyte antigen-DR-mediated diabetic nephropathy progression is a promising therapeutic target. Front. Endocrinol..

[58] Joshi I., Carney W.P., Rock E.P. (2023). Utility of monocyte HLA-DR and rationale for therapeutic GM-CSF in sepsis immunoparalysis. Front. Immunol..

[59] Logt A.-E., Wetzels J.F.M. (2025). Reassessing Rituximab in Membranous Nephropathy: Efficacy, Limitations, and the Path Ahead. Kidney Int. Rep..

[60] Casan J.M.L., Wong J., Northcott M.J., Opat S. (2018). Anti-CD20 monoclonal antibodies: Reviewing a revolution. Hum. Vaccines Immunother..

[61] Tomita A. (2016). Genetic and Epigenetic Modulation of CD20 Expression in B-Cell Malignancies: Molecular Mechanisms and Significance to Rituximab Resistance. J. Clin. Exp. Hematop..

[62] Tipton T.R.W., Roghanian A., Oldham R.J., Carter M.J., Cox K.L., Mockridge C.I., French R.R., Dahal L.N., Duriez P.J., Hargreaves P.G. (2015). Antigenic modulation limits the effector cell mechanisms employed by type I anti-CD20 monoclonal antibodies. Blood.

[63] Counsilman C.E., Zijde C.M., Stevens J., Cransberg K., Bredius R.G.M., Sukhai R.N. (2015). Pharmacokinetics of rituximab in a pediatric patient with therapy-resistant nephrotic syndrome. Pediatr. Nephrol..

[64] Liang H., Deng Z., Niu S., Kong W., Liu Y., Wang S., Li H., Wang Y., Zheng D., Liu D. (2024). Dosing optimization of rituximab for primary membranous nephropathy by population pharmacokinetic and pharmacodynamic study. Front. Pharmacol..

[65] Fogueri U., Cheungapasitporn W., Bourne D., Fervenza F.C., Joy M.S. (2019). Rituximab Exhibits Altered Pharmacokinetics in Patients with Membranous Nephropathy. Ann. Pharmacother..

[66] Merkt W., Lorenz H.-M., Watzl C. (2016). Rituximab induces phenotypical and functional changes of NK cells in a non-malignant experimental setting. Arthritis Res. Ther..

[67] Wlodarczyk M., Torun A., Zerrouqi A., Pyrzynska B. (2024). NK Cell Degranulation Triggered by Rituximab Identifies Potential Markers of Subpopulations with Enhanced Cytotoxicity toward Malignant B Cells. Int. J. Mol. Sci..

[68] Heiber J.F., Geiger T.L. (2012). Context and location dependence of adaptive Foxp3(+) regulatory T cell formation during immunopathological conditions. Cell. Immunol..

[69] Chen Q., Kim Y.C., Laurence A., Punkosdy G.A., Shevach E.M. (2011). IL-2 controls the stability of Foxp3 expression in TGF-beta-induced Foxp3+ T cells in vivo. J. Immunol..

[70] Capuano C., Pighi C., Molfetta R., Paolini R., Battella S., Palmieri G., Giannini G., Belardinilli F., Santoni A., Galandrini R. (2017). Obinutuzumab-mediated high-affinity ligation of FcγRIIIA/CD16 primes NK cells for IFNγ production. Oncoimmunology.

[71] Vo D.-N., Alexia C., Allende-Vega N., Morschhauser F., Houot R., Menard C., Tarte K., Cartron G., Villalba M. (2018). NK cell activation and recovery of NK cell subsets in lymphoma patients after obinutuzumab and lenalidomide treatment. Oncoimmunology.

[72] Lin Y., Han Q., Chen L., Wang Y., Ren P., Liu G., Lan L., Lei X., Chen J., Han F. (2024). Obinutuzumab in Refractory Membranous Nephropathy: A Case Series. Kidney Med..

[73] Sethi S., Kumar S., Lim K., Jordan S.C. (2020). Obinutuzumab is Effective for the Treatment of Refractory Membranous Nephropathy. Kidney Int. Rep..

[74] Hartinger J.M., Kratky V., Hruskova Z., Slanar O., Tesar V. (2022). Implications of rituximab pharmacokinetic and pharmacodynamic alterations in various immune-mediated glomerulopathies and potential anti-CD20 therapy alternatives. Front. Immunol..

[75] Marshall M.J.E., Stopforth R.J., Cragg M.S. (2017). Therapeutic Antibodies: What Have We Learnt from Targeting CD20 and Where Are We Going?. Front. Immunol..

[76] Milcent B., Josseaume N., Petitprez F., Riller Q., Amorim S., Loiseau P., Toubert A., Brice P., Thieblemont C., Teillaud J.-L. (2019). Recovery of central memory and naïve peripheral T cells in Follicular Lymphoma patients receiving rituximab-chemotherapy based regimen. Sci. Rep..

[77] Liu Y., Xu K., Xiang Y., Ma B., Li H., Li Y., Shi Y., Li S., Bai Y. (2024). Role of MCP-1 as an inflammatory biomarker in nephropathy. Front. Immunol..

[78] Xu J., Hu H., Sun Y., Zhao Z., Zhang D., Yang L., Lu Q. (2024). The fate of immune complexes in membranous nephropathy. Front. Immunol..

[79] Stai S., Lioulios G., Christodoulou M., Papagianni A., Stangou M. (2023). From KDIGO 2012 towards KDIGO 2021 in idiopathic membranous nephropathy guidelines: What has changed over the last 10 years?. J. Nephrol..

[80] Logt A.-E., Justino J., Vink C.H., Brand J., Debiec H., Lambeau G., Wetzels J.F. (2021). Anti-PLA2R1 Antibodies as Prognostic Biomarker in Membranous Nephropathy. Kidney Int. Rep..

[81] Teisseyre M., Brglez V., Cremoni M., Fernandez C., Graça D., Boyer-Suavet S., Benzaken S., Esnault V.L.M., Seitz-Polski B. (2023). Risk Factors Associated with the Occurrence of Anti-rituximab Antibodies in Membranous Nephropathy. Clin. J. Am. Soc. Nephrol..

[82] Häusler D., Häusser-Kinzel S., Feldmann L., Torke S., Lepennetier G., Bernard C.C.A., Zamvil S.S., Brück W., Lehmann-Horn K., Weber M.S. (2018). Functional characterization of reappearing B cells after anti-CD20 treatment of CNS autoimmune disease. Proc. Natl. Acad. Sci. USA.

[83] Ling C., Chen Z., Lei L., Xi Y., Zhang H., Wu D., Hua L., Liu X. (2025). Identification of Risk Factors for Relapse following Rituximab Therapy in Children with Steroid-Sensitive Nephrotic Syndrome. Kidney Dis..

